# Remote care in UK general practice: baseline data on 11 case studies [version 2; peer review: 2 approved]

**DOI:** 10.3310/nihropenres.13290.1

**Published:** 2022-11-29

**Authors:** Trisha Greenhalgh, Sara E. Shaw, Anica Alvarez Nishio, Richard Byng, Aileen Clarke, Francesca Dakin, Stuart Faulkner, Nina Hemmings, Laiba Husain, Asli Kalin, Emma Ladds, Lucy Moore, Rebecca Rosen, Sarah Rybczynska-Bunt, Joseph Wherton, Sietse Wieringa

**Affiliations:** 1 Nuffield Department of Primary Care Health Sciences, University of Oxford, Oxford, OXFORDSHIRE, OX2 6GG, UK; 2 Independent Lay Adviser, London, UK; 3 University of Plymouth, Plymouth, PL4 8AA, UK; 4 Nuffield Trust, London, W1G 7LP, UK; 5 Centre for Sustainable Health Education, University of Oslo, Oslo, NO-0316, Norway

**Keywords:** Remote consultations, general practice, digital inclusion, triage, access, video consultations, telephone consultations, e-consultations

## Abstract

**Background:**

Accessing and receiving care remotely (by telephone, video or online) became the default option during the coronavirus disease 2019 (COVID-19) pandemic, but in-person care has unique benefits in some circumstances. We are studying UK general practices as they try to balance remote and in-person care, with recurrent waves of COVID-19 and various post-pandemic backlogs.

**Methods:**

Mixed-methods (mostly qualitative) case study across 11 general practices. Researchers-in-residence have built relationships with practices and become familiar with their contexts and activities; they are following their progress for two years via staff and patient interviews, documents and ethnography, and supporting improvement efforts through co-design. In this paper, we report baseline data.

**Results:**

Reflecting our maximum-variety sampling strategy, the 11 practices vary in size, setting, ethos, staffing, population demographics and digital maturity, but share common contextual features—notably system-level stressors such as high workload and staff shortages, and UK’s technical and regulatory infrastructure. We have identified both commonalities and differences between practices in terms of how they: 1] manage the ‘digital front door’ (access and triage) and balance demand and capacity; 2] strive for high standards of quality and safety; 3] ensure digital inclusion and mitigate wider inequalities; 4] support and train their staff (clinical and non-clinical), students and trainees; 5] select, install, pilot and use technologies and the digital infrastructure which support them; and 6] involve patients in their improvement efforts.

**Conclusions:**

General practices’ responses to pandemic-induced disruptive innovation appear unique and situated. We anticipate that by focusing on depth and detail, this longitudinal study will throw light on why a solution that works well in one practice does not work at all in another. As the study unfolds, we will explore how practices achieve timely diagnosis of urgent or serious illness and manage continuity of care, long-term conditions and complex needs.

## Introduction

The coronavirus disease 2019 (COVID-19) pandemic was (among other things) a unique opportunity for digital innovation in the healthcare sector^1^. As described in more detail in our protocol paper^2^, it triggered unprecedented changes in general practice. Remote digital access (e.g. web portals for booking appointments) and remote (telephone, video and electronic) clinical consultations were technically possible pre-2020, but most primary care staff and patients did not use them (in January 2020, for example, around 14.7% of all general practice consultations in UK were by telephone^3^ and fewer than 1% were by video or e-consultation^4^). In early 2020, UK general practice rapidly introduced remote triage^5^ (by web template or telephone) and remote consulting (mostly by telephone) as the default option. Dramatic changes (chiefly, a shift to online booking and a major increase in the proportion of consultations undertaken by telephone, which increased to over 50%^3^) were achieved at pace and scale but implementation was challenging^4,6–9^ and many patients needed in-person care for clinical or social reasons^4,10^. Between March and August 2020, the absolute number of general practice consultations fell (reflecting covid-related scale-back of services and reduction in demand), but by October 2020 the absolute number of consultations each week was significantly higher than, and the proportion of primary care consultations undertaken remotely was approximately double, pre-pandemic levels^3^. A government policy of ‘remote-by-default’ care was introduced in July 2020 (in the hope that this would increase efficiency of services)^11^ but reversed a few months later^12^ because it was unpopular with patients and concerns had emerged about quality and safety (e.g. missed diagnoses, safeguarding challenges, over-investigation, over-treatment, and threats to the therapeutic relationship), digital inequalities, increased burden on the patient, and increased staff workload and stress^13,14^.

Notwithstanding commendable efforts to make digital services accessible and ensure that the needs of the digitally excluded are met^15,16^, the tendency of digitalisation of services to worsen socio-economic inequalities is well-described^2,17^. Digitally-supported service models are often depicted as more efficient than in-person alternatives, though evidence supporting this claim is sparse even in studies of carefully selected low-risk patients^2^. One large study of telephone-first models in general practice showed an overall *reduction* in efficiency compared to standard access models, but there was wide variation between practices with some reporting benefits and others no change^18^.

Whilst introducing digital innovations in general practice is known to be complex and setting-specific, most previous studies were not designed to produce sufficient descriptive detail to account for differences between practices, nor to explore how services evolve over time (e.g. by purchasing new technologies or uninstalling and disinvesting in failed ones). This study was designed to fill this gap by producing longitudinal, granular descriptions of how practices try to balance remote and in-person care in the current ‘new normal’ (characterised by recurrent waves of COVID-19 and various post-pandemic backlogs), taking account of local and national contextual influences and historical path-dependencies. The purpose of this paper is to present baseline data on the 11 participating practices.

## Methods

**Figure 1:**
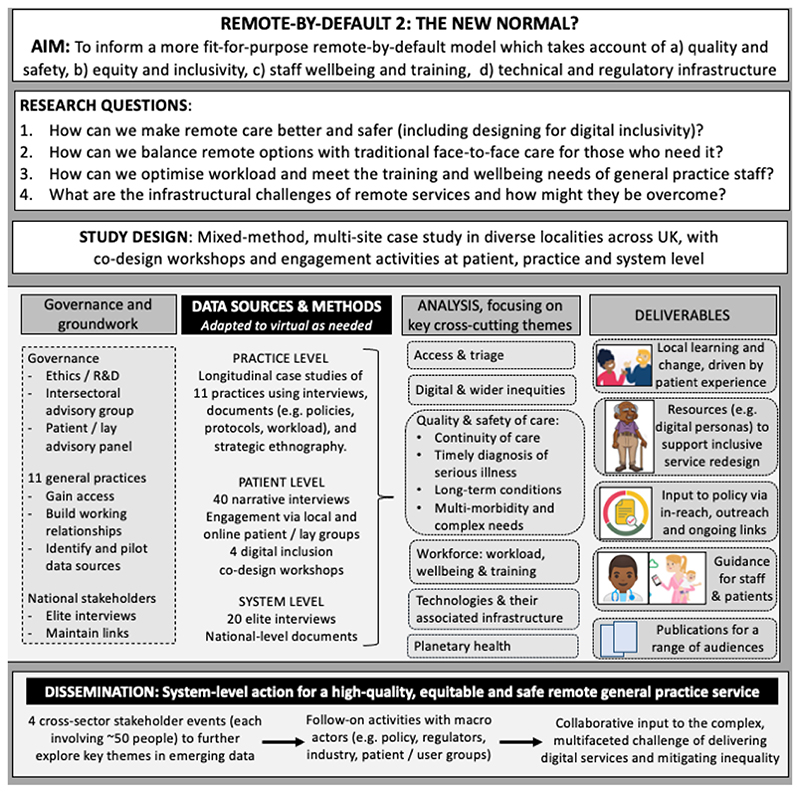
Study flowchart.

Full details of governance, NHS ethics approval and methods are reported separately^2^. Briefly, Remote by Default 2 is sponsored by the University of Oxford and overseen by an independent advisory group with a lay chair and patient representation. Aims, research questions and study design are summarised in Figure 1. Workstream 1 uses an embedded researcher-in-residence^19^ and case study methodology^20^ to develop multi-site longitudinal studies of general practices, using mostly qualitative methods (interviews, ethnography, workshops) but including some descriptive quantitative data as part of the case study narrative (e.g. population demographics). Workstream 2 captures patient experiences and uses co-design with patients and staff to re-imagine service models with a focus on overcoming digital inequalities. Workstream 3 engages national-level stakeholders.

The findings reported here (predominantly from Workstream 1) are based on the interviews and fieldwork summarised in Table 1, collected between October 2021 and June 2022—a period we called the ‘familiarisation process’ in which each researcher-in-residence used publicly available sources (e.g. practice website) and interviews to gain a deep familiarity with a practice’s history, population, ethos and current challenges. This baseline work was conducted under participating practices’ pandemic restrictions (i.e. limited or no in-person visits), hence most of the 127 interviews reported in this paper were conducted by phone or video link. Interviewees were approached initially via the individual staff member who was the practice’s named point of contact with the researcher-in-residence, usually in person. Whilst we tried to conduct formal semi-structured interviews where possible, we found that whereas busy clinicians and support staff were difficult to pin down for formal interviews, they could often fit in a short phone call or engage in an email correspondence (we called these ‘informal interviews’). We had hoped to interview as wide a range of staff as possible in each practice (GPs, nurses, managers, administrative staff) but in some busy practices we used convenience sampling for initial interviews (i.e. whoever had time to speak to us) and plan to balance any uneven sampling at a later date. Interviews lasted between 10 and 90 minutes. The start of fieldwork was delayed in one practice (River Road) because of local ethics and governance sign-offs; another practice (Queens Road) joined the study later than others so these practices have limited data to date. All practice names are pseudonyms.

An additional data source was public-domain documents and web resources describing aspects of each practice (practice leaflets and annual reports, notification boards, telephone answering services, practice booking agenda), plus local census data on population demographics, income, housing, education levels, crime levels and so on and land registry data on housing (e.g. via Streetcheck.co.uk).

As noted in Table 1, background interviews with policymakers provided wider context for remote access and remote care policies in UK general practice.

Interviews were conducted in private with no others present, audiotaped with consent and relevant sections transcribed. Our multidisciplinary team had a total of seven researchers in residence (three academic general practitioners [AK, EL, SW], four postdoctoral social scientists, one of whom originally trained as a nurse [LM, NH, SR-B, JW). All but SW and JW were female, and all had been trained in qualitative research generally and organisational ethnography in particular. They worked in pairs, matching complementary backgrounds (partly so as to balance prior assumptions and biases – e.g. a GP researcher was matched with someone with no clinical background), to manage and thematically analyse data, supported by NVIVO software. Each team produced a familiarisation document of 20-40 pages consisting of a narrative about their practice along with interview quotes and selected quantitative data (e.g. list size, percentage of consultations conducted in person). For each practice, qualitative data were analysed thematically and quantitative data inserted where appropriate to enrich the familiarisation document.

Each researcher-in-residence also took responsibility for exploring a cross-cutting theme using data from all 11 practices, working in dialogue with the team member who was most familiar with each practice. Two senior academic general practitioners [AC and TG] gave feedback on the familiarisation documents, helped researchers refine these where needed, and synthesised an over-arching narrative across the 11 participating practices and all cross-cutting themes. Other team members provided research management support [SF], general overview and support in one locality [RB] and lay insights [AAN]. Online team meetings held weekly or fortnightly allowed informal discussions around themes selected by the researchers-in-residence and allowed them to learn what issues were salient across the 11 sites.

A one-page summary of each practice’s progress so far was shared with the practice named contact and approval gained before inclusion in the *Extended data*^21^. Our external advisory group with patient and lay representation gave feedback on an earlier draft and its chair [AAN] approved the final submission.

The results section below presents the baseline findings from the practices. We have deliberately not given detailed information about staff members when providing quotes so as to protect the confidentiality of informants.

## Results

### Overview of participating practices

In Table 2, we provide a one-paragraph summary of each practice, which we have anonymised. We have classified each practice by index of multiple deprivation (from 1 = most deprived to 10 = least deprived decile) and by digital maturity using the following five-point scale^2^: • (traditional – few or no digital innovations or strategy), •• (traditional with lone innovator – one person keen and attempting to introduce digital innovations and services), ••• (digitally curious – experimenting with digital innovations but not planning or implementing these strategically), •••• (digitally strategic – investing in digital innovations and services, and in some cases strategically *disinvesting* in them) and ••••• (system-oriented – confidently providing a range of digital services and seeking to support others to do the same). Practices’ digital maturity was assessed during 2021 but broadly reflects what was in place (e.g. in terms of knowledge, technical infrastructure and strategic vision) before the pandemic.

As shown in Table 2, the 11 general practices have a wide geographical spread covering inner-city locations in Scotland (Range Park, River Road), remote towns and villages in Wales (Carleon, Rhian), and various settings in England including major cities (Towerhill, Westerly), smaller cities and towns (Newbrey, Camp St, Ogden East, Queens Road) and villages (Fernleigh). Whilst we achieved wide demographic and geographical variation across three jurisdictions, our sample did not include any practices from the north of England or Northern Ireland.

Socio-economic status of the populations served ranges from very deprived rural (Carleon) and urban (Ogden East, River Road, Range Park, Westerly) to fairly affluent rural (Fernleigh) and urban (Towerhill). We deliberately oversampled from deprived localities—for example, whilst one practice (Fernleigh) is in the top decile for Index of Multiple Deprivation, three (Rhian, River Road and Ogden East) are in the bottom decile. Some practices (Camp St, Newbrey, Queens Road) have a very mixed population with some ‘postcode pockets’ of deprivation. The ethnicity of practice populations varies from 35% White with an extremely diverse ethnic mix (Westerly, Queens Road) to 99% White (Carleon); both Welsh practices (Carleon and Rhian) have a high proportion of Welsh-speaking patients and staff. The inner-city practices in Scotland and England (Range Park, River Road, Westerly) have young populations and quite high list turnover; Fernleigh (serving affluent retirement villages) has a more stable population but a high proportion of elderly.

Total practice list sizes vary widely from 31,000 (Camp St) to 2300 (Range Park). List sizes per full-time and salaried partner range from 2300 (Rhian) to fewer than 1000 (Range Park). In terms of staff mix, numbers of nurse practitioners, advanced health care practitioners, nurses and other health care support staff differ widely across practices. Many teams in the English practices are highly multidisciplinary, allowing a high degree of functional flexibility and providing the GPs to focus more on complex cases or specialty interests. The smaller Scottish and the Welsh practices have notably fewer non-medical staff.

Exact numbers of administrative and support staff are hard to capture (in some cases, such data are unavailable; in others, they change frequently). It is clear that some practices have a sophisticated division of labour among reception and support staff as well as among clinicians, and also some well-defined data management and IT support roles (Camp St, Fernleigh, Towerhill and Westerly appear advanced in this regard). In some other practices, the support roles for digital services do not exist at all or are less well developed.

Many practices are involved in non-core activities. Eight of the practices (Carleon, Camp St, Fernleigh, Ogden East, River Road, Rhian, Towerhill, Westerly, Queens Road) are teaching and training practices and two (Fernleigh, Towerhill) were involved in research before joining this study. Some practice members undertake additional activities, for example Carleon clinicians cover a community hospital; Fernleigh is a dispensing practice, and Towerhill partners are involved in local medical politics, organisation and management, with one partner working on the GP Federation board. One Range Park GP is a longstanding member of the Local Medical Committee. These activities suggest that whilst our sample is diverse in many dimensions, most or all are more outward-looking than average.

All practices have many core values in common. Interviewees in every practice, for example, talked of the practice’s commitment to its local population; a desire to provide high-quality, evidence-based, patient-centred care (and in many cases, providing high-quality care was seen as implicit rather than articulated as a value, though safety was occasionally mentioned); to be a happy and cohesive practice team with attention to staff training and wellbeing; to use multidisciplinary and holistic approaches in order to address illness in its social and cultural context; and to minimise inequalities of access and provision. Whilst interviewees in all practices said that continuity of care was valued, many described a trade-off between continuity and (for example) efficiency or timeliness (see ‘cross-cutting themes’ below).

### Innovation and digital maturity

Interviewees from almost all participating practices depicted their organisation as forward-looking and keen to innovate, using terms like “dynamic and positive”, “forward thinking” and “aiming to embrace change”. These comments may reflect the fact that any practice which is prepared to join a research study on digital innovation is to some extent keen to innovate.

Our familiarisation process suggested, however, that practices varied widely in their organisational antecedents for innovation as outlined in a systematic review of the diffusion of innovations literature^22^. This showed that organisations that are able to introduce innovations (should they judge them appropriate) are distinguished by a number of features: structural preconditions (larger size, a flat management structure, devolved decision-making, a clear division of labour and well-differentiated roles, and slack resources that can be [re]deployed), absorptive capacity for new knowledge (a high level of pre-existing knowledge in relevant areas, the skills and systems to capture and distribute knowledge within the organisation, and wide internal and external networks), and receptive context for change (leadership, strategic vision, clear goals, a climate where it is acceptable to take risks, and high-quality data systems to monitor progress). But even when an organisation is able to innovate *in general*, it may assess a potential innovation as a poor fit with its focus and mission (see ‘readiness’ below).

Some practices in our sample (notably, Camp St, Fernleigh, Westerly and Towerhill) appear to have many of the structural and cultural preconditions for innovation. They are large in size, have some slack resources (at least compared to our other practices), and have leadership, strategic vision and clear goals for developing further. They also have high absorptive capacity for new knowledge (with technology-savvy staff, high-quality infrastructure and rich internal and external networks). Some practices possess certain preconditions for innovation but appear to be held back by insufficient slack, heavy workload and (in some cases) outdated premises and systems. As an informant in Newbrey put it “*overwhelming workload* … *leaves no time for innovation*.”

This background helps explain where practices currently lie on our digital maturity scale, which reflects three dimensions of maturity^9^: readiness (strategic alignment, leadership, resources), capability (what is currently installed and possible) and infrastructure (the technological and human infrastructure to support digital innovation).

The Remote by Default 2 practices illustrate the full range of digital readiness. At one end of the continuum, Towerhill has a clear and bold vision to embrace digital innovation and views digital access and consultations as an excellent fit with its strategic goals. At the other end, practices such as Range Park have made a clear strategic decision *not* to prioritise digitally advanced forms of remote care because they do not feel it is currently right for their patients (and perhaps also for their staff). Other practices present a more mixed picture. Newbrey, Queens Road, River Road and Westerly, for example, whose practice populations are socio-economically and ethnically mixed, are progressing cautiously with specific digital innovations while attending carefully to patients who are digitally disadvantaged in various ways.

In terms of digital capability, the practices also represent a wide range (though all have *some* digital capability). All practices are using telephone access and telephone consultations in much greater numbers than before the pandemic, but their telephone systems vary significantly in their dependability and functionality. One limitation is the sheer number of calls the system can cope with. The existing telephone systems in several practices (Camp St, Newbrey, Queens Road, Range Park) are described by staff as at or above capacity, and patients in even more practices describe not being able to get through on the phone. One practice (Towerhill) has recently introduced a sophisticated cloud-based telephony and triage system which will offer greater capacity.

Most practices have the capability to provide online consultations—most commonly using the accuRx add-on for SystmOne. A few have plans to adopt more advanced systems. As with phone, online consultation systems can be more or less sophisticated and have more or less capacity—a technical issue which we address in the cross-cutting theme below.

Many of the practices developed remote systems rapidly in response to COVID-19 and some are pulling back from these, partly because infection control restrictions around in-person consultations have eased. In some practices the changed working patterns inflicted by COVID-19 were very gratefully given up; GPs and nurses had described days spent only on the telephone (“call centre medicine”) as cognitively challenging and unfulfilling. Most practices appear to have continued only with the digital services that appear to add value outside the pandemic context.

A good example of this is video consultations. Only one practice (Range Park) never used video; the remainder had the capability, but many did not persist with it. A few practices (Ogden East, Towerhill, Westerly) are still routinely using video consultations and now have established systems and protocols for targeting them appropriately. Four practices (Carleon, Camp St, Fernleigh and Rhian) initially introduced but then abandoned video, and two more practices (Newbrey, River Road) now use this modality very sparingly (“once in a blue moon” as one previously enthusiastic GP said). In a previous study conducted in the first 18 months of the pandemic, we wrote about the widespread non-adoption and abandonment of the video option in UK general practice^4^.

The quality of infrastructure to support digital services also varies hugely across our sample of practices. At the more advanced end, Towerhill, Fernleigh and Westerly upgraded their infrastructure before the pandemic and were in a strong position to support a move towards more digital services. At the other, Rhian and Range Park are struggling with the most basic infrastructure and cannot make progress (even if they wished to) until this is improved. Scotland had a country-wide initiative to develop the infrastructure for video consultations before the pandemic^7^, though this was to a large extent separate from general IT infrastructure development.

Each practice’s digital maturity is thus a combination of its strategic readiness, its existing capability and its infrastructure. Table 3 shows examples of each level.

In the next section, we describe some themes emerging from our 11 practices with their varied histories, characteristics and patient populations. These include access, triage and capacity; other aspects of quality and safety of care; meeting the needs of the disadvantaged and digitally excluded; staff wellbeing and training; and technologies and their associated infrastructure.

### Cross-cutting themes

Six key themes, described in detail below, were evident in our data:

- *Access, triage and capacity*: problems which patients had accessing care and which practices had managing demand;
- *Other aspects of quality and safety of care*, such as managing risk and achieving continuity;
- *Meeting the needs of the disadvantaged and digitally excluded*—and in particular how the shift to digital triage and remote consulting seemed to exacerbate existing inequities of access;
- *Supporting and training staff and students*, especially the unease which new trainees felt about conducting telephone consultations;
- *Technologies and their associated infrastructure*, especially how practices’ pre-existing technological (e.g. server capacity) and human (e.g. in-house expertise) infrastructure shaped and constrained what innovations were possible to implement;
- *Patient involvement in improvement efforts*, especially (in the context of continuing pandemic restrictions) the difficulty of setting up the processes for patient-led change.

#### THEME 1: Access, triage and capacity

Many GPs in our sample expressed concern about whether they were “*seeing the right populations*”. Whilst they would escalate a situation if they believed there was a clinical need (for example, bringing someone in to be seen in person), there was a concern that they may not always become aware of such need. However, demand is high and rising, and staff in many practices used flooding metaphors (“*swamped*”, “*deluged*”, “*opening the floodgates*”) to describe the volume of telephone calls and digitally-enabled requests for consultations they were having to deal with. This sense of losing (or having already lost) control of the threshold to primary care was palpable in many interviews. Hence, in the current context, we consider access as playing out in tension with capacity.

Every practice sought to avoid the problem of a patient in need being unable to access care. Some practices serving non digitally confident populations (Carleon, River Road, Rhian, Range Park, Westerly) have until recently relied mainly on telephone or walk-up requests that are triaged by reception staff. However, telephone queues—and physical queues at the front door—are becoming unmanageable in these practices. Partly for resource reasons, one or two practices appear to be stuck in a situation where clinicians and support staff are simply working harder (and becoming burnt out) as demand inexorably rises; others have introduced changes (Westerly, and more recently River Road, introduced online consultations; Rhian have hired a new business manager whose brief includes rationalising the appointments system).

Ogden East, also serving a deprived population, uses a different system—no triage by receptionists but a GP-led call-back system to every patient. One GP commented that this system allowed them to practice better care: they felt more in control of the day, reserving more time for more complex patients. They reflected that in such calls, they handled the query differently to how they might have done in an in-person appointment—they could be more candid and (for example) suggest to a patient that they go away and look something up. Ogden East also offer appointments on request to any walk-in patients.

Our baseline data reveals a tendency for online consultations to be used to take the pressure off the front desk. In Ogden East, for example, staff encourage patients to fill out an online consultation request if they are unhappy with the length of time they need to wait for an appointment. Yet several practices (e.g. Camp St, Fernleigh, Newbrey, Ogden East, Rhian) have found that online consultations are inefficient (because they collect irrelevant data, and because some patients use them for problems that staff consider inappropriate, especially if the system is available at night), potentially unsafe (patients have been known to use them for chest pain) and stressful (because of the sheer numbers coming in). In some practices (e.g. Rhian) the capability for online consultations is present but few patients use them (probably because the more traditional telephone and walk-in booking system is accessible and preferred by staff and patients). Online consultations appear to increase clinician workload while relieving workload for support staff (in another flooding metaphor, one GP described having to “*wade through*” multiple online consultation forms).

To control the digital “*floodgates*”, several practices (Fern-leigh, Towerhill, River Road) now restrict the time slots where online consultation forms are available, and other practices are considering doing this. This move was described by one interviewee as a shift from “*demand-driven*” to “*capacity-driven*” provision. It is worth noting that in the practices where such digital gatekeeping is heavily used, support staff shared stories of near-miss cases where patients (typically elderly and with multiple health problems) either gave up trying to contact the practice or attended Accident & Emergency. In response to such critical events, one practice (Fernleigh) plans to move to clinician total triage.

One practice (Camp St) has introduced non-digital triage tools (standard operating procedures, guidance) and trained particular groups of staff in using these. Camp St also operates a ‘safety valve’ system where extra appointments are added to the system once it gets full (usually about 10 am every morning); this practice also has a safety valve for when the duty doctor is overrun, whereby other GPs and advanced clinical practitioners are asked to take up some of the load although this was noted as sometimes causing tension between GPs. Others (e.g. Fernleigh) make selected use of email to communicate with patients and signpost them to the appropriate part of the system.

In Westerly, the reception team have been trained to undertake an initial assessment of appointment requests, advising patients with minor illnesses to contact a pharmacy and booking some patients into appointment slots according to the practices booking rules (e.g. smear requests booked into a nurse in-person appointment; asthma reviews booked into a nurse telephone appointment). There is also practice guidance about booking in-person appointments for patients with selected symptoms (such as abdominal pain). All other appointment requests – whether made by phone, in person or online are allocated a full appointment slot. Short ‘triage’ slots to quickly review an online consultation have not been introduced.

#### THEME 2: Other aspects of quality and safety of care

We were surprised that many aspects of quality and safety of care, including how to achieve timely diagnosis of urgent or serious illness, how best to manage long-term conditions, and how to deliver care for patients with complex needs, were largely absent from our dataset of initial interviews. National policymakers with a safety brief knew of rare examples of ‘never-events’ with possible links to lack of a face-to-face assessment (e.g. death of previously healthy young adult from operable acute abdominal condition), but they emphasised that formal audits of telephone assessments (in which an experienced clinician reviewed an audiotape of the call along with the written record) identified the vast majority as high-quality and safe. It appears that clinicians undertaking telephone assessments have a low threshold for arranging an in-person assessment if indicated—hence the use of telephone by default tends to be *inefficient* rather than *unsafe*.

Practice staff seemed to take quality and safety of care as given, so long as patients could be seen in an appropriate and timely way. For this reason, access and triage (see above) were depicted as mission-critical to quality and safety. The only other aspects of quality and safety which came up repeatedly in our baseline interviews were continuity of care and risks of technology failure.

Continuity was universally depicted as an aspect of high-quality care. However, our interviewees held different views on what continuity was (one-to-one continuity of care, continuity within a small sub-team, or continuity of information), how continuity should be delivered in practice, and the trade-offs against other practice priorities. No practice in our sample had a strict personal list system, but some (e.g. Camp St, Fernleigh) had a ‘usual GP’ arrangement and one (Westerly) operated a ‘buddy group’ system in which GPs and advanced clinical practitioners were clustered in small groups so patients were highly likely to be allocated one of a small number of known clinicians. Queens Road offers patient choice to see a named GP through its online bookable system but encourages patients to see GPs with expertise in specific clinical areas across other practice sites in its medical group. One practice (Tower-hill), which has a mostly young professional population, had tried and abandoned a usual GP system in favour of “*everyone sees everyone*”.

Many practices espoused continuity whilst describing systems that appeared to conflict with the goal of continuity—such as multidisciplinary clinics in which different elements of care are dealt with by different practitioners or appointments that are bookable only on the day. These arrangements had often been introduced to deal with rising demand, with loss of continuity as an unintended consequence.

Most practices operated a triage system for urgent appointments where access to care is prioritised over continuity. Some interviewees suggested that continuity was not as important as patients receiving the “*right*” care and felt that patients would inevitably move “*from one to the other*” practitioner, especially in urgent or high-priority situations. However, continuity was viewed as particularly important for certain patients (especially those with complex needs and multiple long-term conditions).

Westerly have undertaken work to improve continuity, and a GP from Fernleigh described continuity as having a “*positive impact on workload*” since “*tasks are easier if the GPs know the patients well*”.

Whilst many interviewees mentioned risks associated with digital technologies leading to threats to quality of care, we have to date identified few actual examples of these. Risks were most evident when clinicians talked about their attempts to do video consultations (which, as noted above, have been abandoned by most practices). In terms of quality, their concerns were threefold: challenges and time spent setting up the technology (including supporting the patient to do so), technology failure (perhaps due to human error), and poor image quality (reliant on the very variable set-ups which patients had at home—especially if they are using a mobile).

#### THEME 3: Meeting the needs of the disadvantaged and digitally excluded

A strong commitment to meeting the needs of disadvantaged groups was a universal core value across all participating practices. When asked to describe their practice’s ethos, one said it was to “*engage with all sectors of the population*” and another highlighted their practice’s emphasis on “*engagement with hard-to-reach parts of the community*”. Similar phrases were used by staff from ‘deep end’ practices in deprived areas and staff from practices in more affluent areas with postcode pockets of deprivation. In the former case, the entire practice logistics were oriented to serving a predominantly or exclusively deprived community; in the latter case, staff were keen not to orient towards the affluent majority at the expense of the more deprived minority (e.g. a traveller site or poor estate).

Those identified at particular risk of digital inequities were people who were poor, elderly, homeless or in poor accommodation, those with drug or alcohol use problems or who speak limited English or lack full citizenship (e.g. asylum seekers), those who are hard-of-hearing (for phone consultations), or with learning difficulties, or with complex physical or mental health needs. The question practices wrestled with was how to align the aspiration to meet the needs of these groups with the reality of an increasingly digital service.

Disadvantaged patients often have complex needs, with multiple social and health problems (poverty, homelessness, low health and digital literacy, chronic illness, cognitive impairment) exacerbating one another. GPs emphasised that it was important to take a holistic perspective, investing in more challenging aspects of a deprived community: “*Making a difference with the difficult stuff has a knock-on effect within the population.*”

Practices were quick to identify various groups of people who find it hard to navigate the health system and emphasised the efforts to ensure that these patients are made welcome and able to get the care they need.

Access (addressed above) is a major component of the equity agenda—but access comes with an equity trade-off: an increasingly digital orientation makes the practice more accessible for some (often the young, digitally capable and less in need)—but at the cost of making it less accessible to others (particularly, the elderly, those not online and those with complex needs). Even when patients are (apparently) digitally connected, they may be unused to using digital technologies either at all or for their healthcare needs. One practice in a deprived locality (River Road) has been encouraging patients to use online consultations but found that some are not in the habit of checking their messages so may miss the GP’s reply.

As noted above, some practices effectively operated an ‘open door’ policy, allowing patients to walk in and book an appointment then and there. One practice (Ogden East), serving a deprived community, was described by staff as striving to be “*very patient centred. Patients are taken seriously if they suggest their need is urgent even if the support staff feel that the problem is relatively minor*.” In contrast, an interviewee from a practice with a more affluent population with some postcode pockets of deprivation (Newbrey) observed that the people who *want* to see a GP are rarely the ones who *need* to see one, hence being overly responsive to patient demand may paradoxically result in a less “patient-centred” service as the most needy are more likely to be overlooked.

Interviewees described how disadvantaged patients often lack family support and their social networks may be sparse—which means they may lack people they can call on to assist them with digital access. Staff commented that the increased social isolation that came with the pandemic had increased demand for in-person appointments (e.g. from young single mothers). On the other hand, assistance from a family member or friend brings its own challenges of privacy, confidentiality and failed demand.

Our initial interviews identified few examples of practices proactively helping patients acquire digital skills (they lacked the capacity to deliver such support), or of up-and-running digital navigator schemes. In some practices, receptionists sometimes helped patients complete their online consultation forms when they phoned in—though such activity may not be an efficient use of receptionist time. In practices with a high proportion of limited English speakers (Range Park, River Road), community navigators are already employed but it is not yet clear whether or how they are assisting in supporting digital access. One practice (Fernleigh) is considering piloting a ‘digital buddy’ system among its affluent elderly village population (which includes retired professionals), in which more digitally confident patients volunteer to link with and support less confident ones.

Some patients, especially those in the multiple jeopardy of several kinds of disadvantage, may be unable to access any services digitally. Staff in practices serving deprived communities noted that digital is increasingly the default option for secondary care and community services, “*when the default method of access to a service is online, this is a potentially an illegal breach of NHS standards to ensure equitable access.” They sometimes needed to act as advocates for their patients to overcome digital barriers in other sectors (e.g. a mental health referral service in which the patient must complete a web registration form to enter the system)*.

#### THEME 4: Supporting and training staff and students

In the context of a wider staffing crisis affecting the UK NHS in general and general practice in particular (especially in deprived areas)^23^, a major contributor to staff morale is workload and the changing division of labour. As noted above, workload is high in all practices and some practices (Newbrey, Queens Road) feel it is at, or close to unsustainable levels. Interviewees attributed some of this workload to factors other than the move to digital (rising patient demand, unfilled staff posts, task-shifting from secondary care). But they considered some as resulting directly from digital options which, far from making work more streamlined and efficient, have made it less so. Online consultations in particular were widely viewed as stressful and inefficient.

Practices are taking various approaches to reducing the very high levels of workload. Attempts to closing the “*digital flood-gates*” by reducing availability of appointments or restricting the time window of the online consultation service was covered under theme 1 above. Another approach is optimising the division of labour within the practice.

The larger practices in our sample (Camp St, Fernleigh, Newbrey, Towerhill, Westerly) have a high degree of disciplinary diversity including GP partners, salaried GPs, advanced clinical practitioners, physician assistants, paramedics, nurses, healthcare assistants, clinical pharmacists, dispensers, and a range of administrative and managerial support staff (including specific staff to support digital innovation in one practice). A wide range of additional staff often allows the GPs to be “*freed up to take a more supervisory role*” (Towerhill interviewee). This “freeing up” also releases the GPs to take on training roles, deal with the more complex and difficult cases, take on executive roles in running the practice and bringing in new innovations, or undertake more outward-facing responsibilities such as working on local medical political groups or practice consortia. Non-medical clinicians and GP trainees look after the more straightforward patients such as those needing long term condition management or with acute minor illness. Dedicated support staff – e.g., the practice manager or senior administrator—oversee the routine running of the practice. All GP partners, however, were still undertaking ‘normal’ day-to-day GP work as well, taking their turn in seeing emergencies and doing booked surgeries.

This kind of advanced division of labour in larger practices illustrates why an organisation’s size is such a strong predictor of its ability to innovate^22^. Apart from some community link worker roles, the smaller practices in Scotland and Wales (e.g. River Road, Range Park, Carleon), were less able to benefit from this multidisciplinary model of working—perhaps for historical or geographical reasons, or because there was less support to develop it in those regions. Carleon, however, has employed paramedics and a pharmacist from England working remotely.

Many interviewees described or alluded to low staff morale. In particular, they described all day remote telephone consulting as having a negative effect on their wellbeing. They missed the team element and seeing each other and the chance to interact with their patients in real life. There was a general sense that practices are currently in a state of flux—they feel that there is no going back to where they were before the pandemic but they have not yet reached a steady state (i.e. a way of running the practice that feels like a sustainable form of business-as-usual).

Some interviewees even depicted staff wellbeing as a safety issue. One GP, for example, started a discussion of future practice plans by saying that the most important thing was to “*keep everybody safe – implementing the workflow plans to keep staff and patients safe*” (practice name omitted to increase confidentiality). This interviewee described ongoing difficulties in staffing for GPs (salaried and partner), nurses and receptionist.

A national training lead expressed grave concern for wellbeing of trainees and young GPs who worked remotely from home and did not have the benefit of ‘corridor consultations’ to discuss concerning cases; one organisation had introduced ‘virtual coffee breaks’ where doctors working remotely could emulate this kind of mutual support and collaborative learning in the digital space.

Many of the problems of staff wellbeing in our participating practices centre around access and triage, in the sense that whoever is bearing the brunt of digital and wider access appears to be suffering (either receptionists are struggling with jammed overloaded telephone lines or GPs are wading through online consultations). Workload increased when patients over-use a system (e.g. sending in multiple requests because they have not yet heard back)—even for understandable reasons. One interviewee commented that remote consultations had “*added a level of complexity*” for receptionists, and in several practices (e.g. Westerly, Fernleigh), turnover of reception staff has increased.

Triage was one of the most contentious and problematic areas contributing to lowered staff morale and wellbeing. As noted above, systems for dealing with triage varied, and in several practices these were undergoing change. Some practices triaged patients as they telephoned the practice—working out if a patient needed face to face consultation or could have a phone call or an appointment with another service—whereas some used total telephone triage (i.e. call-backs) for all patients. Systems where a decision had to be made about modality sometimes seemed to generate staff stress and further problems down the line. Because of high demand, staff rarely had the time or headspace to channel the patient to the most efficient route to care. As one interviewee (in Fernleigh) said, “*whereas pre-pandemic the team were trying to signpost to other available community services (such as the minor eye casualty service or minor injury unit etc) now they have mostly given up and just find any available appointment they can*”.

At this stage, we have limited data on education and training in relation to increased use of digital technologies and services, although 8 of the 11 practices are training practices. A few interviewees described how students, trainees or early-career clinicians were finding it difficult to become skilled and confident with so much clinical practice happening remotely. With telephone consultations in particular, trainees felt they were very much “*on their own*”; the physical arrangements meant that they could no longer easily drop in on a next-door consulting room or start an informal corridor or tea-room conversation to discuss cases. Rather, specific plans had to be made for trainees to be able to debrief on patients. We will be exploring this sub-theme in more detail as the study unfolds.

#### THEME 5: Technologies and their associated infrastructure

Star defined infrastructure as “*what other things run on*” (including both technological components such as wires and servers, and also the human, organisational and regulatory ‘scaffolding’ that puts technologies in place and supports their use)^24^. She observed that a feature of infrastructure is that it is generally backgrounded but becomes visible on breakdown. In this study, the wider technological and regulatory infrastructure was most noticeable by its relative absence—for both good and bad reasons.

In terms of policy, all practices in our sample appear to have been strongly encouraged and supported to adopt digital technologies and offer remote services during the pandemic. Development of digital access and digital consultations as a long-term strategy was largely in response to a national “remote by default” policy directive made in July 2020^11^, but a more recent reversal of this (pressure from the new Secretary of State for Health to revert to “in-person by default”^12^) had a mixed reception from GPs and their staff, since it cut across the changes to digital services that practices had been working to achieve.

We detected widespread unease about what changes might be about to happen at the political and policy level (where, broadly speaking, GPs and their work is perceived as undervalued). In a letter to patients Queen Road explained that “*A sustained attack by the media on general practice and the seemingly ill-informed demands of the health secretary for more face – to – face appointments have left us demoralised, broken and burnt out*.” Some of the practices (especially those perhaps nearest to the policy process) expressed heartfelt concerns about the future of general practice more generally—with near-unsustainable workload, a workforce crisis and apparent government inaction. In Towerhill, one GP said that their involvement in the local primary care network “*helps grow the standing of the practice*,” suggesting that it acts as a “*hedge*” allowing the practice to deal with what politicians “*throw at general practice.*”

Health information infrastructures are also patchworked and path-dependent, in which components emerge incrementally and so cannot be installed or replaced wholescale^25^. The varied fortunes of the 11 practices illustrate how pre-existing technological infrastructure (the ‘installed base’ in Star’s terminology^24^) both enabled and constrained remote consultation and triage practices. Prevailing infrastructural arrangements led to the selection and use of particular technologies and the development of particular routines, processes, knowledge and workarounds, which in turn set the organisations on a particular infrastructural path going forward.

As noted above, most case sites initially introduced video communication platforms that had been specifically developed for medical consultations (Attend Anywhere and accuRx), but did not persist with this modality. Whilst these bespoke products have been designed to align with clinic workflows (e.g. ‘virtual waiting area’ to help manage the flow of patients attending their virtual appointments) and information governance requirements (e.g. avoid the need for patients to download software or provide personal information), implementation has been limited by network connection problems (at the practice and/or patients’ homes), difficulties interfacing with electronic records, a lack of adequate audio-video equipment and private space in the clinic, and the time involved to set up and troubleshoot the technology—all of which potentially jeopardise the professional standards of care, risk and workforce capacity described above.

Practices that have continued to use video are generally characterised by a strategic investment in IT and material infrastructure (e.g. clinic and office room set up, dual screens to view video alongside patient records), targeted use of the modality (with a clear understanding of how and when video would add value), local knowledge and skill to use and support each other with the technology, a degree of technical integration across video and electronic record applications (specifically accuRx and SystmOne), and the careful alignment of clinic workflows with software functionality (e.g. to book video appointment slots and support real-time video connection when deemed necessary by the clinician).

Whilst the telephone is an old technology, the extended use of telephone for triage and consultations depends on both traditional (‘legacy’) systems and also new or extended systems which (for example) allow patients to send digital photos and documents. Clinicians talked about how these technical adjuncts, alongside new clinical and communication skills, have reshaped their perceptions of the potential role of this medium in clinical care. Some sites have developed (and others are considering) advanced telephony systems, such as wifi connecting phones with headsets (Fernleigh) and phone call recording and cloud storage (Westerly).

Our initial interviews have highlighted the infrastructural work that has gone into creating and embedding new work processes and routines. Organisational routines are defined as “*recognisable, repetitive patterns of interdependent action carried out by multiple actors*”^26^. Routines are situated within a socio-material context—in other words, the interdependent actions of human actors are structured around time, physical spaces, and material and technological artefacts^27^. Participants described the challenges in distributing and coordinating administrative and clinical tasks, and the emergence of ‘hidden’ or ‘invisible’ articulation work (defined as work that is necessary for dealing with anticipated contingencies, but which is not formalised or documented^28^), in order to support and accommodate the technology. For example, reception staff at Rhian routinely print out emails and other electronic messages from patients, and transfer the paper documents to a physical in-tray in the office, thereby aligning old and new systems of collaborative working.

Another example of articulation work is now receptionists in traditional practices such as River Road complete the online appointment request forms on behalf of patients (on the other end of the phone) who are unable or unwilling to use the online system themselves. Such work contributes significantly to ensuring that remote consulting and triaging practices are ongoing and feasible (‘keeping the show on the road’), though it is not known how much time is spent in this way nor how it increases or decreases the efficiency of the triage system in different settings. Our ongoing research will seek to understand this kind of articulation work in the busy setting of general practice reception areas and back offices.

In addition to the patient access and digital exclusion issues described in theme 3 above, staff interviews have also highlighted usability and access problems more generally. Sometimes staff have been able to address basic design flaws in subtle but important ways. For example, Westerly saw a significant increase in the use of online consultation requests after they updated the practice website to make it easier for patients to navigate and locate the electronic forms.

However, service teams are often unable to change or reconfigure the technical aspects of the system because they lack technical knowledge, IT support and relevant permissions. For example, many online consultation templates are considered too long and burdensome, with much redundancy and repetitive questioning, but these cannot be altered by the practice. A number of interviewees highlighted design flaws within their telephony systems for managing call queues. For example, in River Road, one of the main reasons for introducing the ‘Footfall’ online booking system was to address patient frustration (and clinical risk), as one nurse explained: “*We don’t have any control over the phone lines. So people were phoning in, and there wasn’t a message to let them know, like they were in the queue. It just rang and rang and rang. People weren’t aware that they were in a queue, and so they were just phoning and hanging up, because they did not think anyone was answering. They were getting very frustrated. They thought we were just sitting here, having tea and coffee and not answering the phone…*.”.

The availability and affordability of technology has also been shaped by funding and procurement decisions, including commercial contracts and professional standards. For example, Clinical Commissioning Groups (CCGs) in England have provided funding for GPs to use accuRx, and government-funded initiatives in Scotland and Wales focused on the roll out of Attend Anywhere in primary care, as part of the pandemic response. Procurement processes remain challenging, with a limited range of solutions that may not be fit for purpose at either end of the digital spectrum. Camp St Group are currently awaiting a CCG funding decision to use a new AI-driven triage and patient flow management system, called KLINIK. They are experiencing this process to be slow and uncertain.

#### THEME 6: Patient involvement in improvement efforts

Although some practices solicit feedback from their patient groups, our preliminary interviews have shown that in general these groups either do not exist or have a demographic (e.g. retired professionals) that is atypical of the practice’s population. There is only informal and ad hoc data on how patients are finding the new systems, the level of patients’ own technical capabilities and how they match the systems that are being offered. One or two practices are undertaking small-scale studies on this topic, and our own research includes a workstream on patient and public involvement.

## Discussion

### Summary

Our in-depth case studies of a diverse sample of 11 general practices have illustrated both commonalities and differences in their approach to digital services. Practices vary in their enthusiasm for and uptake of such services. However almost all struggle with access and demand and with how to ensure that they are prioritising and meeting the needs of vulnerable and disadvantaged patients. These practices represented a wide range of settings and challenges but the sample is not intended to represent the totality of general practice or convey a ‘typical’ picture.

Research and surveys undertaken by others in 2021-2022 affirmed a number of findings of this familiarisation phase, including problems of accessing primary care^29^; substantially increased workload in UK primary care^30^; perceptions by GPs of increased clinical risk with some remote consultations^31,32^ or when patients are encouraged to monitor their own chronic conditions such as blood pressure and report by telephone^33^; exacerbation of inequities, especially among the most vulnerable groups, as services went digital^34–40^; a primary care workforce that is increasingly stressed and underconfident^41–44^; multiple infrastructural challenges to establishing remote services during the pandemic^45–48^; and challenges to patient input to research efforts during the pandemic^49^.

### Themes to explore further

We will be taking forward some high-level issues (listed below) which have emerged from our previous work and our work so far in this study.

#### Patient input to practice change efforts

The limited input of patients to the design and evaluation of digitally supported services was striking, and due (we surmise) largely to pandemic-related restrictions on meetings and the high levels of workload and staff stress in many practices (there is simply no slack to undertake patient consultations). We hope that the co-design component of this study (described in our protocol paper^2^) will help to bring patient-centredness in improvement initiatives more to the fore.

#### Efforts to improve the triage process

Triage seems to lie at the heart of much concern over workloads, stress, staffing and staff morale. Several practices have recently changed their triage system and others plan to shortly. It is already apparent that there is no one-size-fits-all triage system, but we hope to tease out what is likely to work for whom, in what kind of circumstances.

#### Efforts to reduce inequalities

Practices are at an early stage in various efforts to support those who may potentially be excluded as services go digital. Our in-depth study design will enable us to explore intersectionality—how different social determinants (e.g. being elderly and poor and chronically sick) combine and interact to worsen digital inequalities. We will also be undertaking co-design activities using digital personas to support efforts to overcome these inequalities.

#### Quality of clinical care

Our early interviews did not pick up on much in the way of comments about what actually goes on in the consultation, nor many specific comments about quality and safety when managing long-term conditions, early indicators of serious conditions (which might be missed in the absence of an in-person encounter), and patients with communication challenges or complex needs). Previous research by ourselves and others suggests that remote care may compromise the therapeutic relationship and continuity of care, lead to more transactional forms of clinical interaction, fewer ‘doorknob consultations’, and delayed diagnosis of serious illness (see our protocol paper for literature review^2^). These problems are likely to affect the patient population disproportionately and generate new kinds of inequity.

Whilst remote assessment may have unacceptable risks for complex and vulnerable patients, it may be convenient and safe (and be associated with better uptake) for routine follow-up of patients with stable long-term conditions. However, there is a danger that if such reviews are undertaken by text messaging, the patient becomes (predominantly at least) an online entity, with adverse impacts on the therapeutic relationship and missed opportunities for key hands-on clinical checks (e.g. foot pulses in diabetes).

#### Selection and procurement of digital technologies

It was evident from our early interviews that some technologies introduced at the height of the pandemic have subsequently been abandoned because they were unfit for purpose and in some cases worsened the problems they were introduced to solve. The early pandemic was a time of relaxing red tape and bypassing regulatory approvals^6^, and governance (financial and clinical) now needs to be fully restored across the NHS. The procurement process for new technologies in the NHS is not always well-aligned with business cycles^50^.

#### Technical functionality

It was clear from our interviews and other data-gathering that the ‘same’ technology (a telephone system, an online consultation system) can have very different functional characteristics depending on the precise product used, which functions have been enabled (or disabled), which local infrastructure it interfaces (or fails to interface) with, the demands placed on it, and human factors such as confidence, training and informal support to use it. With few exceptions, technological resources and know-how were greater in larger practices. As noted in theme 5 above, legacy infrastructure and contracts signed in the past sometimes created path dependencies which prevented practices from upgrading or replacing digital technologies in the ways they would have liked to. We will explore such issues in ethnography and digital walk-throughs as the study progresses.

#### Reverting to a more in-person model of care?

As the pandemic recedes, practices are re-evaluating the benefits of the digital-by-default technologies and ways of working that they adopted in early 2020. Many are now in flux. They have clearly been through a huge change and a process of destabilisation, and are now searching for a sustainable way forward in the longer term. Again, there is no one-size-fits-all model but we hope to support and describe some ways of gaining an effective balance of traditional and digital forms of care.

#### Support for small practices

The apparent dependence of successful digital services on a sophisticated division of labour, and the latter’s dependence on practice size, raises important questions about critical mass going forward—either small practices are destined to become obsolete or different ways must be found to support them.

#### Planetary health

This theme did not come up in our early interviews for this study, but has featured in our previous research and we will be actively exploring it in future interviews. Travel to healthcare appointments generates greenhouse gases. Remote service provision could potentially reduce this, though carbon savings in primary care may be modest as patients live locally, and could be achieved at the expense of waste (e.g. over-diagnosis, over-treatment or over-referral). Local savings (of various kinds) may come at the expense of ‘hidden’ environmental waste.

## Conclusion

We are living through a period of great change in general practice. Our study in depth and detail of 11 diverse practices has illustrated the unique, situated and creative ways in which GP practices have dealt with rapid technological innovation and major changes in service delivery. We have identified a number of key issues to take forward in our ongoing work, which includes in-person ethnography of both clinical and administrative work.

## Figures and Tables

**Table 1:** Data sources for baseline findings reported in this paper.

Source	Formal interviews	Other (e.g. informal non-audiotaped phone calls, emails, preliminary site visits)	Total interviews
Camp St Group	8 GPs, 2 pharmacists, 3 managers, 1 patient	2 informal practice visits with brief chats to 8 staff (GPs, support staff, GP trainee)	14 formal 8 informal
Carleon	1 GP, 1 manager	Brief chats with 2 GPs and 1 manager	2 formal 3 informal
Fernleigh	2 GPs, 11 patients	3 managers, 1 nursing lead	13 formal 4 informal
Newbrey	1 GP, 1 manager, 2 care coordinators	2 informal practice visits with brief chats to 4 staff	4 formal 4 informal
Ogden East	3 GPs, 1 paramedic, 1 nurse practitioner, 1 manager, 1 support staff, 1 patient	-	8 formal
Queens Road	0	3 GPs, 1 manager	0 formal 4 informal
Range Park	1 (GP)	1 (GP)	1 formal 1 informal
Rhian	1 GP, 1 GP trainee, 3 managers, 1 pharmacist, 1 advanced nurse practitioner, 1 practice nurse	Brief chats with 3 GPs and 1 manager	8 formal 4 informal
River Road	1 nurse, 1 healthcare assistant, 3 managers	3 GPs	5 formal 3 informal
Towerhill	3 GPs, 1 GP trainee, 1 nurse, 3 support staff (some more than once)	7 informal visits with brief chats to ~10 staff	11 formal (with 8 staff) 10 informal
Westerly	2 GPs, 1 former trainee, 1 nurse, 1 manager, 2 receptionists	4 hours ethnographic observation in back office, and attending a 1-hour clinical staff meeting	8 formal
National stakeholders	12 (11 policymakers, 1 clinical training expert)	-	12 formal
GRAND TOTAL	-	-	86 formal 41 informal

**Table 2:** Summary characteristics of participating practices.

Practice	Description
**Camp St Group** Deprivation decile: 6^th^ Digital maturity •••• List size: 31,000	This group practice is in a southern English commuter town, about 20 miles from a major city. It spans three sites; all share one computer system. There are 15 partners and 40 additional staff including paramedics, one advanced clinical practitioner, six nurses, four pharmacists, four healthcare assistants and many support staff. Their population is ethnically and socio-economically diverse with affluent and low-income populations living in adjacent streets. Various digital innovations have been piloted and some but not all have been retained.
**Carleon** Deprivation decile: 2^nd^ Digital maturity • List size: 7500	This rural practice covers three sites in north Wales. Each site serves a different demographic—from picturesque retirement villages close to a National Park, farming communities to some of the most deprived boroughs in Wales. There are 5 part-time GP partners, two registrars, and relatively few attached staff (two advanced nurse practitioners and two practice nurses) plus a pharmacist working remotely from England. Carleon is a teaching and training practice and is responsible for a community hospital. Digital provision is currently limited (and restricted to telephone), partly because both staff and patients seem to prefer traditional in-person appointments. However, demand is high and rising and whilst the current system is described as “not really working” (hence there is some tension for change), the direction of change is not yet clear.
**Fernleigh Medical Group** Deprivation decile: 9^th^ Digital maturity ••• (approaching ••••) List size: 15,000	This well-resourced 7-partner dispensing practice in central southern England serves a mainly affluent and elderly population. The staff of 40 includes 7 salaried GPs with many staff in non-medical clinical roles, freeing the GPs for more complex patients and extended roles. The practice prides itself for being innovative in terms of both processes and technology, and for being involved in a range of non-core activities such as training, undergraduate teaching, research and working with the local community. Many of these mainly elderly patients are not comfortable using online contact methods.
**Newbrey Surgery** Deprivation decile: 9^th^ Digital maturity: between •• and ••• List size: 21,000	This suburban practice lies on the outskirts of a university city in central England, with 5 GP partners. A staff of around 30 includes 9 salaried GPs, one advanced clinical practitioner, six nurses, two paramedics, one social prescriber, one care co-ordinator, several healthcare assistants and a large administrative team. It serves a relatively affluent population (young professionals, healthcare workers and their families), but also has some postcode pockets of deprivation. The practice struggled recently with unmanageable demand, experienced telephone triage as inefficient (because of doublehandling), and has introduced a proactive patient booking team aimed at giving as many patients as possible their preferred appointment type.
**Ogden East** Deprivation decile: 1^st^ Digital maturity: • List size: 8300	This practice serves a deprived borough in a city in western England. It has two full-time GP partners and five salaried GPs, plus a wide a range of other clinical staff and administrative staff. It is a teaching and training practice. 88% of patients are white; many are in the lowest socio-economic decile and include homeless people and travellers. A high proportion have complex co-morbidities and many have low health and digital literacy. The practice provides a drug and alcohol service. They strive to be patient-centred and allow patients to select their preferred appointment type. They have introduced some digital modalities (including online consultations which they found generated high workload). They are keen to avoid digital exclusion of vulnerable patients. Plans include introducing some kind of remote triage.
**Queens Road** Deprivation decile: 7^th^ Digital maturity: ••• List size: 13,000	The practice, in a small city in Western England, has a mixed socio-demographic and serves a high number of people with refugee status. It has the highest usage of the telephone interpreting service Language Line in the region. It has two GP partners and four salaried GPs, with a range of allied clinical and administrative staff. It provides a drug and alcohol service and a chronic pain clinic that offer non-medical solutions to patients on prolonged opiate use. The practice has multiple routes of access (online booking system, telephone, and an online consultation platform). It works flexibly around the needs of patients with known vulnerabilities (i.e. homeless and people with learning disabilities) by enabling them to make appointments at the front desk and offering in-person double appointment slots. There is an expectation that patients without additional needs will adapt to the remote triage system. These changes are met with some resistance by patients (and staff perceive some hostility) but the practice believe that digitisation of services for the majority will help meet rising patient demand.
**Range Park** Deprivation decile: 1^st^ Digital maturity: • List size: 2300	This small practice in a major city in central Scotland has two GP partners, an attached community link worker and a district nursing service. It serves a population that is 88% white with high socio-economic deprivation, low health literacy and many young families. Patients have high levels of illness and comorbidities linked to social determinants, with high rates of drug and alcohol use. The lead GP, who is active on local and national primary care committees, has a longstanding presence in the community and knows many patients and families well; consequently, she is confident managing many consultations by telephone. Receptionists make triage decisions. The practice has no plans to expand its digital services. Rather, its priorities are to improve outreach and support to the local population through non-digital means.
**Rhian** Deprivation decile: 3^rd^ Digital maturity: •• (aspiring to •••) List size: 11,500	This teaching and training practice in a small south Wales town has a village branch surgery three miles away. There are five GP partners, two salaried GPs, four nurses, two healthcare assistants and an on-site pharmacist. Some staff have been there over 20 years, though several partners are currently on breaks or soon to retire. The population is relatively deprived; it includes a former coal mining community as well as a growing number of young professional families relocating to a large new housing estate and retired people. Patients can currently ask for their preferred consultation type, which is triaged by receptionists. Rhian was an early digital adopter 20 years ago but more recently has fallen behind (see ‘Innovation and digital maturity’). The new business manager is keen to make changes, bring people with him, rework the staffing structure and appointment systems, and find digital solutions that “click together”.
**River Road** Deprivation decile: 1^st^ Digital maturity: ••• List size: 5500	This practice (list size 5,500) serves a young and ethnically diverse population in a very deprived borough on the outskirts of a major city in southern Scotland. The practice is housed in a modern complex that includes a library, leisure centre and various social services. There are four part-time GP partners, one nurse, one healthcare assistant, one community link worker, and aligned health visitors and district nurses—but no advanced nurse practitioners, paramedics or pharmacists. It is a teaching and training practice and strongly committed to serving the local community. Many patients have complex needs and low health literacy. Recent introduction of e-triage has greatly reduced stress among reception staff but added to the GPs’ workload.
**Towerhill** Deprivation decile: 8^th^ Digital maturity: •••• (approaching •••••) List size: 15,800	This four-partner teaching and training practice is sited in a fairly affluent borough in a major city in south-east England. It has three salaried GPs, five physician assistants, an advanced clinical practitioner, a pharmacist, three business managers and various administrative staff. Partners are active in the Clinical Commissioning Group, Primary Care Network and GP Federation. The practice ethos is contemporary—a young, ethnically diverse and digitally-savvy group of GPs (“we’re interested in the new and shiny”) serving a population with a similar demographic. Staff value quality of care and evidence-based practice. They are keen to innovate and embrace change, and they enjoy state-of-the-art premises, IT infrastructure and numerous digital technologies. A high proportion of consultations occur remotely. Patients may only contact the practice online; those who telephone are talked through a digital template. However, some support staff are concerned that the less digitally literate patients are being overlooked, and have shared stories about patients ending up in Accident and Emergency because they could not access the practice.
**Westerly** Deprivation decile: 2^nd^ Digital maturity: ••• List size: 27,000	This large teaching and training practice lies on the outskirts of a major city in southern England. There are six GP partners, six salaried GPs, two registrars plus 30 staff including 7 nurses, two pharmacists, three managers, and a large and well-differentiated reception and support team. Historically, the practice served a deprived population but the area is rapidly becoming more socio-economically mixed due to extensive property building in the area. It is very ethnically diverse; many patients are limited English speakers and there is a high use of the Language Line interpreting service. There is a strong emphasis on continuity of care. Access is primarily by telephone and the NHS app, through which patients can book slots for telephone or in-person appointments. There is also a daily walk-in clinic. The emphasis is on patient choice rather than strict triage. However, telephone contact is currently very high; reception staff are stressed and feel that demand is unsustainable. Current priorities are rationalising the appointment system, replacing the phone system, and addressing staff wellbeing.

**Table 3:** Examples of practices at different levels of digital maturity.

Digital maturity level	Examples
• Traditional	Range Park and Carleon both offer traditional forms of access and consultation types, which are considered more appropriate to the deprived populations served. For this reason, they take a reactive approach to policy initiatives that push the digital agenda. Both practices have mainly phone access, and with digital access either not available or not used. Despite modern premises, the IT infrastructure at Range Park is weak (“even the phone connection can be poor...”—GP).
•• Traditional with lone innovator	Rhian was an early digital adopter (one of the first practices in the country to have its own website, set up by the lead GP who is a keen digital innovator) but is now described by staff as lagging behind neighbouring practices, partly because of major infrastructural challenges (unsuitable premises dating from the 1970s and a legacy IT system that interfaces awkwardly with video and online consultation technologies and does not support analytics). Current technologies include MyHealthOnline, an online platform used for prescriptions and appointment bookings (which is not used much), and the My Surgery App which has a link on the surgery website and helps patients contact the surgery (there are plans to increase use). Rhian is thus quite advanced in terms of capability (digital technologies are installed) but lacks infrastructure and strategic readiness (perhaps chiefly because they are awaiting a move to new premises).
Between •• and •••	Newbrey was described by some interviewees as at level 2 but assessed by the researcher in residence as at or approaching level 3 (digitally curious). During the pandemic they adopted a telephone-first system for every patient with a very limited number of in-person consultations and a resulting adverse effect on clinician morale. A lone innovator GP attempted a video consultation service, but this did not catch on among their partners. The practice now makes widespread use of the accuRx online consultation tool and there is a GP-led triage system.
••• Digitally curious	Ogden East is trying out digital innovations but not yet using these as part of a fully-developed strategy. It has introduced a number of digital innovations which are in fairly widespread use. The practice has a telephone first triage system—all patients are initially assessed over the phone for need for further intervention. The practice has capability to use accuRx for video consulting but use has diminished since pandemic restrictions eased. Patients can book appointments through SystmOne or can complete a telephone call-back request online consultation form from the website, but most patients book their appointments by telephone. At Queens Road, staff view digital services as offering a potential solution to managing the demand from patients but they also express concern about unfettered access from easily-accessible online consulting platforms. There are multiple entry points into the practice (telephone, bookable appointments through the NHS app, accuRx online consulting platform; and in-person for people that cannot reach them remotely). Telephone lines are exceptionally busy and the accuRx platform is switched off when the practice reach capacity. Despite multiple access routes, in the last patient survey (2021) only 43% of patients were satisfied with access. River Road is currently experimenting with a system called Footfall for triage. Footfall is similar to other online consultation systems which patients can access on the practice website and which allows them to write down their presenting complaint and receive a reply from the practice by secure email. This is reported as having taken the pressure off reception phone lines. This practice initially started using video consulting using the Scotland-wide Attend Anywhere platform, but abandoned it because of poor infrastructure (inadequate computer and phone quality—both in the practice and in many patients’ homes). GPs have reverted to using pictures sent as phone or email attachments. Westerly had introduced a range of digital services prior to the pandemic and a few GPs had already trialled video consulting and accuRx. Because of concerns about digital exclusion when introducing online access and consultations, they reviewed several different providers and asked a small group of patients to test out two different services before choosing a system which included a voice-activated option preferred by some patients. However, this system was not funded by the Clinical Commissioning Group and was not used much by patients pre-pandemic, so a year later they switched to eConsult which was available fully funded. During the pandemic, they developed a new practice website and adopted a new telephony system to cope with vastly increased pressure on the phone lines.
•••• Digitally strategic	Camp St Group rated their own digital maturity as level 3 (one partner reflected that they are “not very sophisticated”), but there are signs that they are already at level 4—not because they have all the latest gadgets but because digital technologies are used creatively and strategically. They have used email for a long time to correspond with patients and are also now using online consultations (Systm1 accuRx) They use an automated response system to encourage people to use online consultations and have introduced workflows with staff who are trained to manage and triage the online consultations. They plan to invest in a new telephony system, aiming “to smoothe demand across the day”. Video was tried but withdrawn as a strategic choice because it was felt to meet very few patients’ needs. As in many practices, the GPs and support staff find online consultations frustrating (as the templates ask too many unrelated questions), so there is a novel plan to introduce a symptom sorter underpinned by artificial intelligence (Klinik). Fernleigh, an early adopter of technologies, use a range of approaches, including telephone triage and telephone consultations (with an in-person consultation arranged if required), accuRx text messaging and email, as well as eConsult online triage forms. Whilst video consultations are possible through accuRx and were used at the height of the pandemic, they are rarely used now (perhaps because most patients are elderly and have relatively low confidence with digital technologies).
•••• System-oriented	Towerhill is at or approaching level 5. It is strategically ambitious (with further digital developments a clear priority) and has very advanced infrastructure and a high level of digital capability—for example, use of the accuRx system for photography, patient text messaging and sending documents. Towerhill was an early adopter (and beta tester) of various digital tools and platforms, and is involved in digital health research (e.g. a locality data warehousing project which has an attached data analyst who can produce ‘dashboards’ on hospital admissions, referrals and more). The practice uses Teams for internal communication and health information exchange (hospital, mental health). They have appointment book interoperability using EMIS which allows them to book patients into linked community clinics (e.g. for ulcer dressings). Several partners are working to support digital innovation beyond the practice—for example, driving the introduction of a cloud-hosted telephone system (X-on) and a long-term condition management system across the Primary Care Network. Their Primary Care Network employs digital champions who are financially supported by the NHS.

## Data Availability

Selected data on this ongoing, mainly qualitative study will be made available to researchers on reasonable request to the lead author. The reason we have not provided full transcripts for all interviews and copies of field notes is that the study design precludes this. We have carefully built relationships with each of the 11 practices based on personal contact from a researcher-in-residence, and worked extensively with staff to build trust and assure the confidentiality of information shared. Our raw data contains highly sensitive information (e.g. receptionists may be fearful that a GP or practice manager in their own practice might read negative things they have said; GPs may have voiced concerns about the commitment of trainees or vice versa). Whilst these raw data will inform our emerging understanding of each individual practice and also the cross-cutting analysis of all practices, we have an over-riding duty to the participants to keep these transcripts confidential. A breach of this duty would not only be unethical but could lead to the practice withdrawing from the study. Our NHS ethics approval is based on assurance of confidentiality of material disclosed by staff members and patients in the practices. For this reason, the only data available to be *publicly* shared is summaries of the practice familiarisation documents that have been approved by the practices. However, it may be appropriate for experienced researchers in this field to seek particular additional data from the corresponding author whose email address is given above, and any such request will be treated on its merits. Mendeley Data: Remove by Default 2. https://doi.org/10.17632/cx6v6zkp49.1^21^ This project contains the following extended data: - Appendix.docx (summary versions of practice familiarisation documents.) Data are available under the terms of the Creative Commons Attribution 4.0 International license (CC-BY 4.0).

## REVISED: Amendments from Version 1

As requested by the reviewers we’ve added additional detail on the background (% of consultations held by video and phone in UK practices) and also in the discussion (references to other research).

**Any further responses from the reviewers can be found at the end of the article**

## Plain english summary

We describe early results from the Remote by Default 2 study, which is following 11 UK general practices for two years as they introduce various kinds of remote appointment booking and clinical consultations. We have been using interviews and ethnography (watching real-world activities), and analysing documents (such as practice reports and websites) to prepare case studies of the 11 practices, which vary widely in size, ethos, geographical location, practice population and digital maturity.

Our initial interviews identified the following cross-cutting themes, which showed both commonalities and differences across the 11 practices:

- *The ‘digital front door’* (patients gaining access using digital portals), which was used to a greater or lesser extent in all practices; some found these systems frustrating and inefficient.
- *Quality and safety*. Staff were concerned about the risk of missing an important diagnosis when consulting remotely, and felt that digitisation could threaten continuity of care.
- *Digital inclusion*. All practices were keen to ensure that patients who lacked digital devices or skills were not disadvantaged; this goal was achieved in different ways (and to different degrees) in different settings.
- *Staff support and training*. Some practices are finding current workload unsustainable due to (among other things) rising patient demand, unfilled staff posts, a post-pandemic backlog of unmet need, and task-shifting from secondary care. Digitisation appears to have increased workload in most practices.
- *Technologies and infrastructure*. The IT infrastructure in each practice had grown in a particular way over time, and was in this sense ‘path-dependent’ (hence, not easily changed).

In conclusion, different practices are responding to the ‘disruptive innovation’ of digital technologies in very different ways, reflecting their different practice populations, settings and priorities. We plan to follow the above themes over time and explore additional themes including the experience and role of patients.

## Open Peer Review

**Current Peer Review Status:** ✔ ✔

**Version 2**

Reviewer Report 06 December 2022

https://doi.org/10.3310/nihropenres.14479.r28880

**© 2022 Dowell A.** This is an open access peer review report distributed under the terms of the Creative Commons Attribution License, which permits unrestricted use, distribution, and reproduction in any medium, provided the original work is properly cited.

✔ **Anthony (Tony) Dowell**^iD^

Department of Primary Health Care and General Practice, University of Otago Wellington, Wellington, New Zealand

I thank the author for their thoughtful and considered responses to review. This is an important ongoing area of research for Primary Care.

***Reviewer Expertise:*** Evaluation of COVID response. Complexity and Implementation science in primary care.

**I confirm that I have read this submission and believe that I have an appropriate level of expertise to confirm that it is of an acceptable scientific standard**.

**Version 1**

Reviewer Report 08 November 2022

https://doi.org/10.3310/nihropenres.14414.r28789

**© 2022 Newbould J.** This is an open access peer review report distributed under the terms of the Creative Commons Attribution License, which permits unrestricted use, distribution, and reproduction in any medium, provided the original work is properly cited.

✔ **Jennifer Newbould**

Cambridge Centre for Health Services Research, RAND Europe, Cambridge, UK

This article reports on a mixed methods, primarily qualitative study, which seeks to study general practices as they balance remote and in-person care in the period following the COVID-19 pandemic. The plain English summary provides a concise and appropriately detailed account of the research with appropriate use of lay language and explanation of more technical terms. The introduction provides good background to the topic area and context within which the data were gathered. In the methods section the authors detail the conduct of the study, with appropriate reference to other documents should the reader require more detailed information. The approach it outlined in detail, including the composition of the team, a point of great importance with the researcher in resistance approach. The results section provides detailed information on the participating practices which provides important context to the findings presented. The clarification of practices by the digital maturity matrix is a helpful way of contextualising where on a spectrum practices are. There are good use of tables and figures throughout this paper which provide detailed information to the reader which enhances the main text. In the discussion section an appropriate summary is provided and themes to be explored further in the study outlined.

In relation to points where further clarification from the authors might be sought:

○ I was unclear if the practice names are actual names or pseudonyms? Clarification on this point might usefully be added to the text.
○ The research is described as mixed methods, primarily qualitative yet this paper describes only qualitative approaches - where there also quantitative elements? Or are these at other time points in the programme of work?
○ It would be useful to clarify for the reader some information on the depth of fieldwork undertaken to inform this paper, as well as the number of interviews conducted.
○ In table 1 the authors identify formal and informal interviews, it would be good to explain within the text how these differ from each other.
○ The results (under innovation and digital maturity) refer to a familiarisation process - it would be beneficial to provide detail to the reader as to what this includes.
○ The sample of 11 practices display characteristics which make the 'non typical' of general practices across England - I feel this caveat to data should be made more explicitly in the discussion section.

Overall, I find this work to be of good quality and endorse the work is clearly presented, of sound study design and the conclusions adequately supported by the results. At a time of enormous change in general practice this feels like a timely and much needed research study, the design of which enables longitudinal and in-depth understanding of a range of practices as they balance remote and in-person care. I wish the authors all the best with the study.

**Is the work clearly and accurately presented and does it cite the current literature?**

Yes

**Is the study design appropriate and is the work technically sound?**

Yes

**Are sufficient details of methods and analysis provided to allow replication by others?**

Yes

**If applicable, is the statistical analysis and its interpretation appropriate?**

Not applicable

**Are all the source data underlying the results available to ensure full reproducibility?**

Yes

**Are the conclusions drawn adequately supported by the results?**

**Yes**

***Reviewer Expertise:*** Primary care research focused on non face-to-face approaches

**I confirm that I have read this submission and believe that I have an appropriate level of expertise to confirm that it is of an acceptable scientific standard.**

Author Response 21 Nov 2022 **Trisha Greenhalgh**, University of Oxford, Oxford, UK Jennifer Newbould (R2) This article reports on a mixed methods, primarily qualitative study, which seeks to study general practices as they balance remote and in-person care in the period following the COVID-19 pandemic. The plain English summary provides a concise and appropriately detailed account of the research with appropriate use of lay language and explanation of more technical terms. The introduction provides good background to the topic area and context within which the data were gathered. In the methods section the authors detail the conduct of the study, with appropriate reference to other documents should the reader require more detailed information. The approach it outlined in detail, including the composition of the team, a point of great importance with the researcher in resistance approach. The results section provides detailed information on the participating practices which provides important context to the findings presented. The clarification of practices by the digital maturity matrix is a helpful way of contextualising where on a spectrum practices are. There are good use of tables and figures throughout this paper which provide detailed information to the reader which enhances the main text. In the discussion section an appropriate summary is provided and themes to be explored further in the study outlined. In relation to points where further clarification from the authors might be sought:

I was unclear if the practice names are actual names or pseudonyms? Clarification on this point might usefully be added to the text.

**Pseudonyms! Added (page 10)**.

The research is described as mixed methods, primarily qualitative yet this paper describes only qualitative approaches - where there also quantitative elements? Or are these at other time points in the programme of work?

**Quant data are descriptive only (e.g. number of patients on list, waiting times).**
**Explained, page 9**.

It would be useful to clarify for the reader some information on the depth of fieldwork undertaken to inform this paper, as well as the number of interviews conducted.

**This was summarised in Table 1 but we've also added a sentence in the main text, page 9**.

In table 1 the authors identify formal and informal interviews, it would be good to explain within the text how these differ from each other.

**This is explained in detail in the protocol paper but added briefly on page 9-10.**

The results (under innovation and digital maturity) refer to a familiarisation process - it would be beneficial to provide detail to the reader as to what this includes.

**Added, page 9**.

The sample of 11 practices display characteristics which make the 'non typical' of general practices across England - I feel this caveat to data should be made more explicitly in the discussion section.

**Added, page 30, but see above - I think we may have over-emphasised this. Actually I think the spread of practices is pretty typical with the exception that we failed to recruit practices who were actually already on their knees from the current NHS pressures. We got a few who weren't far from that state though**. Overall, I find this work to be of good quality and endorse the work is clearly presented, of sound study design and the conclusions adequately supported by the results. At a time of enormous change in general practice this feels like a timely and much needed research study, the design of which enables longitudinal and in-depth understanding of a range of practices as they balance remote and in-person care. I wish the authors all the best with the study. **Thanks! We build on your work of course**. ***Competing Interests:*** n/a

Reviewer Report 17 August 2022

https://doi.org/10.3310/nihropenres.14414.r28742

**© 2022 Dowell A.** This is an open access peer review report distributed under the terms of the Creative Commons Attribution License, which permits unrestricted use, distribution, and reproduction in any medium, provided the original work is properly cited.

？ **Anthony (Tony) Dowell**^iD^

Department of Primary Health Care and General Practice, University of Otago Wellington, Wellington, New Zealand

As stated by the authors in the abstract, 'virtual care', became the prime form of providing primary care, during the early stages of the corona virus pandemic, providing opportunities for technology innovation and also challenges for both patients and health providers in maintaining effective clinical relationships and care. As restrictions on face to face health interactions have been relaxed, health systems are weighing up the optimal balance between virtual and 'in person' contact.

Primary care and General Practice are notable for the variation in working style and practice across both individual practitioners and practices. This study with its depth longitudinal qualitative case study and co-design approach has the potential to provide valuable insights as to how this balance of different means of care will be negotiated.

**Abstract:**

○ The abstract provides a clear and concise review of the background and methodology chosen to answer the research questions.

**Introduction:**

○ The introduction provides a clear background to the UK general practice response to the need for a 'digital response' at the start of the COVID-19 pandemic, and provides appropriate descriptors of the different types of virtual care offered.
○ It would be helpful (if available), particularly for international readers, to give some indication of the balance of digital portal and video / telephone consult use prior to the pandemic.
○ The introduction appropriately highlights the importance of equity considerations in the provision of digital care, and questions to view that digital equates to efficient care delivery. The authors make a compelling case for both the need for their study and the chosen methodology by highlighting the absence of detailed in depth and highly granular case studies to explore how different practices will navigate the new 'hybrid' environment.

**Methods:**

○ The overall mainly qualitative methodology is appropriate to answer the research questions. Particular strengths of the methodology are the use of the 'researcher in residence' in stream one and the co-design elements of workstream 2.
○ The paper states that most of the interaction with the practice team was via phone or video link, which seems to imply the researcher in residence was not able to observe many of the day to day workings of the practice. Was this the case?
○ Table 1 is a useful graphical summary to the aims and methodology.
○ There is variation in the interview sampling frame in each practice. Was there contact with a representative with all those who would be undertaking virtual health consultations in each practice. E.g., at least one nursing interview?
○ The use of additional information sources and document analysis adds to the rigour of the methods.
○ It is clear that the research team has appropriate training in the chosen research methods. To what extent were there specific group training sessions for the whole team, such that there were consistent practice overviews?
○ The use of the two senior researchers providing a synthesised overarching narrative and feedback on the familiarisation documents is a useful addition to the methods.

**Results:**

○ The results and major findings are clearly defined and displayed.
○ The Table 2 summary is very helpful.
○ While the protocol paper contains details of the digital maturity scale, it may be helpful to give a brief idea, particularly for international readers, what the different levels mean. It would be helpful to indicate at the start that Table 3 gives and indication of these levels.
○ The use of widespread geographical variation is a positive feature of the study. Given the multiple commentaries about the 'North / South' divide, was there any reason why there were no North of England practices in the study?
○ The practice selection descriptions do seem to suggest 'all are more outward-looking than average'. Was there any attempt made, either by incentivisation or other means, to try and recruit more 'average' practices to the study?
○ The initial finding of practice commitment to the local population is an important finding from an appreciative inquiry perspective. This could perhaps be highlighted more prominently?
○ I am not entirely clear from the results how quickly pre-pandemic preparedness was translated into a digital COVID response or whether the levels of digital maturity in Table 3 represents largely their digital status prior to COVID? Interview data was collected between October 2021 and June 2022; were interviews and data intended to assess digital opportunities and challenges during that time frame?
○ The results section flows well in a logical sequence from the description of the different capacity and capability to the cross cutting themes.
○ The results describe primary care services under pressure (flooding metaphors) and the pressure of having to respond digitally. It would be helpful to make clear whether there was any reduction in overall workload from patients staying away because of COVID concerns or other reasons, hence freeing up clinician time to undertake digital consultations? This has been noted in other settings, with one concern being potential harm because patients delayed seeking usual care.
○ The cross cutting themes are well described with appropriate focus on access, quality and access.
○ There is a wealth of detail in the cross cutting themes. It might be helpful to have a box / table with a list of the overview themes and brief descriptor at the outset to guide the reader through this section.

**Discussion:**

○ The discussion provides a concise summary of findings and then places further themes for exploration in context.
○ The discussion themes are well written. There is no external referencing in the discussion either with other local UK or international commentaries. Is there a reason for this, given the comparative literature on such themes as digital response to COVID, co-design and equity in these areas?

**Is the work clearly and accurately presented and does it cite the current literature?**

Yes

**Is the study design appropriate and is the work technically sound?**

Yes

**Are sufficient details of methods and analysis provided to allow replication by others?**

Yes

**If applicable, is the statistical analysis and its interpretation appropriate?**

Yes

**Are all the source data underlying the results available to ensure full reproducibility?**

Yes

**Are the conclusions drawn adequately supported by the results?**

Yes

***Reviewer Expertise:*** Evaluation of COVID response. Complexity and Implementation science in primary care.

**I confirm that I have read this submission and believe that I have an appropriate level of expertise to confirm that it is of an acceptable scientific standard, however I have significant reservations, as outlined above**.

Author Response 21 Nov 2022 **Trisha Greenhalgh**, University of Oxford, Oxford, UK Tony Dowell (R1) As stated by the authors in the abstract, 'virtual care', became the prime form of providing primary care, during the early stages of the corona virus pandemic, providing opportunities for technology innovation and also challenges for both patients and health providers in maintaining effective clinical relationships and care. As restrictions on face to face health interactions have been relaxed, health systems are weighing up the optimal balance between virtual and 'in person' contact. Primary care and General Practice are notable for the variation in working style and practice across both individual practitioners and practices. This study with its depth longitudinal qualitative case study and co-design approach has the potential to provide valuable insights as to how this balance of different means of care will be negotiated.
**Abstract:**

○ The abstract provides a clear and concise review of the background and methodology chosen to answer the research questions.

**Introduction:**

○ The introduction provides a clear background to the UK general practice response to the need for a 'digital response' at the start of the COVID-19 pandemic, and provides appropriate descriptors of the different types of virtual care offered.
○ It would be helpful (if available), particularly for international readers, to give some indication of the balance of digital portal and video / telephone consult use prior to the pandemic.

**This has been done (page 8). Answer: In January 2020 14.7% of primary care consultations were by phone; in April 2020 this had risen to 50.6% and by October 2020 had fallen again to 37%. The absolute number of consultations fell dramatically in the early weeks of the pandemic: from 22 million per week in Jan 2020 to 15 million in April - but rose to almost 27 million in October. Video and e-consultations accounted for a tiny percentage pre-pandemic and didn't increase much percentage-wise during the pandemic**.
**In England, for example, video and e-consultations combined accounted for fewer than 0.5% of general practice consultations in December 2021**

○ The introduction appropriately highlights the importance of equity considerations in the provision of digital care, and questions to view that digital equates to efficient care delivery. The authors make a compelling case for both the need for their study and the chosen methodology by highlighting the absence of detailed in depth and highly granular case studies to explore how different practices will navigate the new 'hybrid' environment.

**Methods:**

○ The overall mainly qualitative methodology is appropriate to answer the research questions. Particular strengths of the methodology are the use of the 'researcher in residence' in stream one and the co-design elements of workstream 2.
○ The paper states that most of the interaction with the practice team was via phone or video link, which seems to imply the researcher in residence was not able to observe many of the day to day workings of the practice. Was this the case?

**It was for the first few weeks when COVID-19 restrictions were in place, which is where we were when the baseline findings paper was submitted. More recent data on all practices includes direct ethnography. We've clarified on page 9 and 32.**

○ Table 1 is a useful graphical summary to the aims and methodology.
○ There is variation in the interview sampling frame in each practice. Was there contact with a representative with all those who would be undertaking virtual health consultations in each practice. E.g., at least one nursing interview?

**No. See Table 1. In some practices (e.g. Queens Road, which came on board a bit later than all the others), we'd only done 'informal' interviews at this stage. We got who we could, as practices were so busy. However, many of these interviews were rich and detailed, and we got a good picture of what the issues were. We wanted to publish this baseline data even though incomplete, precisely because it forms the baseline against which we have followed their progress.**

○ The use of additional information sources and document analysis adds to the rigour of the methods.
○ It is clear that the research team has appropriate training in the chosen research methods. To what extent were there specific group training sessions for the whole team, such that there were consistent practice overviews?

**Not so much group training sessions but weekly or fortnightly 'huddles' - online themed discussion sessions where we learn what's going on in the other practices and what approaches are being used. Added, page 11.**

○ The use of the two senior researchers providing a synthesised overarching narrative and feedback on the familiarisation documents is a useful addition to the methods.

**Results:**

○ The results and major findings are clearly defined and displayed.
○ The Table 2 summary is very helpful.
○ While the protocol paper contains details of the digital maturity scale, it may be helpful to give a brief idea, particularly for international readers, what the different levels mean. It would be helpful to indicate at the start that Table 3 gives and indication of these levels.

**The problem here is we're going to be repeating the protocol paper if we add detailed explanations about the digital maturity scale. We already have this brief explanation: "... digital maturity using the following five-point scale ^2^ : • (traditional - few or no digital innovations or strategy), •• (traditional with lone innovator - one person keen and attempting to introduce digital innovations and services), ••• (digitally curious - experimenting with digital innovations but not planning or implementing these strategically), •••• (digitally strategic - investing in digital innovations and services, and in some cases strategically** *disinvesting* in them) and ••••• (system-oriented - confidently providing a range of digital services and seeking to support others to do the same)."
**Note to reviewer: the protocol paper and the baseline findings paper are intended to be read as a pair. They are both published on NIHR Open site and will be linked to each other. We wanted to name the same reviewers for both papers but NIHR Open didn't allow this because of a blanket rule that the reviewers should be "independent". This makes no sense of course in our case but we don't make the rules!**

○ The use of widespread geographical variation is a positive feature of the study. Given the multiple commentaries about the 'North / South' divide, was there any reason why there were no North of England practices in the study?

**Just coincidence really. We're just applying for another grant to extend RBD2 and we've added a site in Birmingham. I guess the point is, Glasgow and South Wales are also pretty deprived and remote Wales also has other features we wanted to sample (e.g. remoteness! And the Welsh language).**

○ The practice selection descriptions do seem to suggest 'all are more outward-looking than average'. Was there any attempt made, either by incentivisation or other means, to try and recruit more 'average' practices to the study?

Point taken, but I wonder if we over-egged this point in the initial draft. All we're saying is that in the current climate (NHS general practice is *on its knees*), ANY practice that agrees to participate in this kind of research is probably atypical. It would of course be impossible to recruit a practice that is too busy to participate (and that's probably most of them). So the only way round this is to interpret the findings in the light of the likely skew. There is however huge diversity in practice characteristics - small/large, affluent/deprived, digitally mature/immature, urban/rural etc etc. I don't think we should over-interpret this sentence "practices were more outward-looking than average". Ethics rules would not allow us to "incentivise" (e.g. pay more than the going rate) to induce participation.

○ The initial finding of practice commitment to the local population is an important finding from an appreciative inquiry perspective. This could perhaps be highlighted more prominently?

**Possibly, but I also wonder if it's just something that all GP practices say? We'll bear it in mind as we follow them further!**

○ I am not entirely clear from the results how quickly pre-pandemic preparedness was translated into a digital COVID response or whether the levels of digital maturity in Table 3 represents largely their digital status prior to COVID? Interview data was collected between October 2021 and June 2022; were interviews and data intended to assess digital opportunities and challenges during that time frame?

Digital COVID response from UK general practice was staggeringly fast—happened over 3 weeks in March-April 2020. The shift was threefold: a) 'total triage' (patients had to book online or wait their turn to have a receptionist help them by phone); b) vast majority of consultations by telephone; c) video consultations introduced using novel (fast-developed) technologies. But the first two of these didn't require much in the way of new tech. What was more important in digital maturity terms was the practice's *strategic* approach to technologies in general. Was there a budget line for technologies in the annual business plan? Were technologies purchased and evaluated *strategically* to pursue particular goals (or were they the pet gadgets of the techy partner)? Was there a training budget for staff to be taught to use tech? Was the practice's IT infrastructure adequate (e.g. server power)? Was there a member of staff around who knew how to install stuff and could parley with helpdesk people? So it's not just 'did they do video consultations?' but 'were they innovative technologically [including acknowledging that tech needs training and support and routinization]?'. To answer the question, digital maturity mostly reflects the status prior to covid (we've clarified this on page 12). None of the practices experienced fundamental transformation during covid, they just switched to online booking and phone call-backs!

○ The results section flows well in a logical sequence from the description of the different capacity and capability to the cross cutting themes.
○ The results describe primary care services under pressure (flooding metaphors) and the pressure of having to respond digitally. It would be helpful to make clear whether there was any reduction in overall workload from patients staying away because of COVID concerns or other reasons, hence freeing up clinician time to undertake digital consultations? This has been noted in other settings, with one concern being potential harm because patients delayed seeking usual care.

**Yes massively, but only for a few months in 2020. Added (page 8). (plus please see response to R2)**

○ The cross cutting themes are well described with appropriate focus on access, quality and access.
○ There is a wealth of detail in the cross cutting themes. It might be helpful to have a box / table with a list of the overview themes and brief descriptor at the outset to guide the reader through this section.

**Great suggestion. Added, page 21-22.**

**Discussion:**

○ The discussion provides a concise summary of findings and then places further themes for exploration in context.
○ The discussion themes are well written. There is no external referencing in the discussion either with other local UK or international commentaries. Is there a reason for this, given the comparative literature on such themes as digital response to COVID, co-design and equity in these areas?

**There was no reason! Some external references now added (thanks for the prompt!).**
***Competing Interests:*** N/A

**Comments on this article**

**Version 2**

**Reader Comment 05 Dec 2022**

**Eleanor Levy**, ARC KSS, Kent Surrey and Sussex, UK

The issues regarding accessibility for digital methods vary depending on local policies and system infrastructure including back office. In my experience, many clinicians have not received any training to familiarise themselves with digital methods and assume that the system adopted early in the pandemic by NHS, MS Teams, offers the same features to patients and the public as it does for their local NHS team. Accessibility online is not usually given the same attention as it would receive if a meeting at a venue were being arranged. As it happens I was excluded from some prearranged NHS regional training because the host could not enable subtitles in Zoom. I now have to wait three months for that training to be delivered via MS Teams, with no assurance that accessibility will be enabled any better via that platform. My GP only offers appointments by phone, resulting in my exclusion. This is simply unacceptable. If it were a commercial organisation, I would be challenging them, as I have done for financial services, energy supply and Broadcasting and streaming services. NHS principles demand better response towards accessibility, a stance which this research tends to support.

## References

[1] Gkeredakis M, Lifshitz-Assaf H, Barrett M (2021). Crisis as opportunity, disruption and exposure: Exploring emergent responses to crisis through digital technology. Information and Organization.

[2] Greenhalgh T, Shaw S, Alvarez Nishio A (2022). Protocol: Remote care as the ‘new normal’? Multi-site case study in UK general practice [version 1; peer review: 1 approved]. NIHR Open Res.

[3] Fraser C, Fisher R (2021). How has the COVID-19 pandemic impacted primary care?.

[4] Greenhalgh T, Ladds E, Hughes G (2022). Why do GPs rarely do video consultations? qualitative study in UK general practice. Br J Gen Pract.

[5] NHS England (2020). Advice on how to establish a remote ‘total triage’ model in general practice using online consultations.

[6] Shaw SE, Hughes G, Wherton J (2021). Achieving Spread, Scale Up and Sustainability of Video Consulting Services During the COVID-19 Pandemic? Findings From a Comparative Case Study of Policy Implementation in England, Wales, Scotland and Northern Ireland. Front Digit Health.

[7] Wherton J, Greenhalgh T, Shaw SE (2021). Expanding video consultation services at pace and scale in Scotland During the COVID-19 Pandemic: National Mixed Methods Case Study. J Med Internet Res.

[8] Murphy M, Scott LJ, Salisbury C (2021). Implementation of remote consulting in UK primary care following the COVID-19 pandemic: a mixed-methods longitudinal study. Br J Gen Pract.

[9] Greenhalgh T, Rosen R, Shaw SE (2021). Planning and Evaluating Remote Consultation Services: A New Conceptual Framework Incorporating Complexity and Practical Ethics. Front Digit Health.

[10] Turner A, Scott A, Horwood J (2021). Maintaining face-to-face contact during the COVID-19 pandemic: a longitudinal qualitative investigation in UK primary care. BJGP Open.

[11] Hancock M (2020). The Future of Healthcare (speech, 30th July).

[12] NHS England (2021). Updated standard operating procedure (SOP) to support restoration of general practice services.

[13] Rosen R, Wieringa S, Greenhalgh T (2021). Clinical risk in remote consultations: findings from in-pandemic qualitative case studies. Brit J Gen Pract.

[14] Wieringa S, Neves AL, Rushforth A (2022). Safety implications of remote assessments for suspected COVID-19: qualitative study in UK primary care. BMJ Qual Saf.

[15] NHS England (2020). Implementing phase 3 of the NHS response to the COVID-19 pandemic.

[16] NHS Digital (2020). How we can support digital inclusion.

[17] Veinot TC, Mitchell H, Ancker JS (2018). Good intentions are not enough: how informatics interventions can worsen inequality. J Am Med Inform Assoc.

[18] Newbould J, Exley J, Ball S (2019). GPs’ and practice staff’s views of a telephone first approach to demand management: a qualitative study in primary care. Br J Gen Pract.

[19] Marshall M, Pagel C, French C (2014). Moving improvement research closer to practice: the Researcher-in-Residence model. BMJ Qual Saf.

[20] Stake R, Denzin NK, Lincoln YS (1994). Handbook of Qualitative Research.

[21] Greenhalgh T (2022). Remote by Default 2. [Dataset] Mendeley Data.

[22] Greenhalgh T, Robert G, Macfarlane F (2004). Diffusion of innovations in service organizations: systematic review and recommendations. Milbank Q.

[23] Nussbaum C, Massou E, Fisher R (2021). Inequalities in the distribution of the general practice workforce in England: a practice-level longitudinal analysis. BJGP Open.

[24] Star SL (1999). The ethnography of infrastructure. Am Behav Sci.

[25] (1998). Changing irreversible networks.

[26] Feldman MS, Pentland BT (2003). Reconceptualizing organizational routines as a source of flexibility and change. Adm Sci Q.

[27] Swinglehurst D, Greenhalgh T, Myall M (2010). Ethnographic study of ICT-supported collaborative work routines in general practice. BMC Health Serv Res.

[28] Hampson I, Junor A (2005). Invisible work, invisible skills: interactive customer service as articulation work. New Technol Work Employ.

[29] NHS England (2022). GP Patient Survey 2022.

[30] Health and Social Care Committee The Future of General Practice.

[31] Johnsen TM, Norberg BL, Kristiansen E (2021). Suitability of video consultations during the COVID-19 pandemic lockdown: cross-sectional survey among Norwegian general practitioners. J Med Internet Res.

[32] Segal JB, Dukhanin V, Davis S (2022). Telemedicine in primary care: qualitative work towards a framework for appropriate use. J Am Board Fam Med.

[33] Neves AL, Li E, Gupta PP (2021). Virtual primary care in high-income countries during the COVID-19 pandemic: Policy responses and lessons for the future. Eur J Gen Pract.

[34] Howells K, Amp M, Burrows M (2022). Remote primary care during the COVID-19 pandemic for people experiencing homelessness: a qualitative study. Br J Gen Pract.

[35] Shah DA, Sall D, Peng W (2022). Exploring the role of telehealth in providing equitable healthcare to the vulnerable patient population during COVID-19. J Telemed Telecare England.

[36] Yu E, Hagens S (2022). Socioeconomic Disparities in the Demand for and Use of Virtual Visits Among Senior Adults During the COVID-19 Pandemic: Cross-sectional Study. JMIR Aging.

[37] Kaihlanen AM, Virtanen L, Buchert U (2022). Towards digital health equity - a qualitative study of the challenges experienced by vulnerable groups in using digital health services in the COVID-19 era. BMC Health Serv Res.

[38] Schenker RB, Laguna MC, Odisho AY (2022). Are we reaching everyone? A cross-sectional study of telehealth inequity in the COVID-19 pandemic in an urban academic pediatric primary care clinic. Clin Pediatr (Phila).

[39] Mueller BR, Lawrence S, Benn E (2022). Disparities in telehealth utilization in patients with pain during COVID-19. Pain Rep.

[40] Huang J, Graetz I, Millman A (2022). Primary care telemedicine during the COVID-19 pandemic: patient’s choice of video versus telephone visit. JAMIA Open.

[41] Jefferson L, Holmes M (2022). GP workforce crisis: what can we do now?. Br J Gen Pract.

[42] Turner A, Morris R, Rakhra D (2022). Unintended consequences of online consultations: a qualitative study in UK primary care. Br J Gen Pract.

[43] Coenen L, Poel LV, Schoenmakers B (2022). The impact of COVID-19 on the well-being, education and clinical practice of general practice trainees and trainers: a national cross-sectional study. BMC Med Educ.

[44] Ansell S, Read J, Bryce M (2020). Challenges to well-being for general practice trainee doctors: a qualitative study of their experiences and coping strategies. Postgrad Med J.

[45] Ftouni R, AlJardali B, Hamdanieh M (2022). Challenges of Telemedicine during the COVID-19 pandemic: a systematic review. BMC Med Inform Decis Mak.

[46] Galvin E, Desselle S, Gavin B (2022). Patient and provider perspectives of the implementation of remote consultations for community-dwelling people with mental health conditions: A systematic mixed studies review. J Psychiatr Res.

[47] Vasireddy S, Wig S, Hannides M (2022). Technical factors can impact on remote consultations in rheumatology: results from a service evaluation during the COVID-19 pandemic. Rheumatol Int.

[48] Wherton J, Greenhalgh T, Hughes G (2022). The Role of Information Infrastructures in Scaling up Video Consultations During COVID-19: Mixed Methods Case Study Into Opportunity, Disruption, and Exposure. J Med Internet Res.

[49] Adeyemi I, Sanders C, Ong BN (2022). Challenges and adaptations to public involvement with marginalised groups during the COVID-19 pandemic: commentary with illustrative case studies in the context of patient safety research. Res Involv Engagem.

[50] Greenhalgh T, Fahy N, Shaw S (2018). The Bright Elusive Butterfly of Value in Health Technology Development Comment on “Providing Value to New Health Technology: The Early Contribution of Entrepreneurs, Investors, and Regulatory Agencies”. Int J Health Policy Manag.

[51] Flyvbjerg B (2006). Five misunderstandings about case-study research. Qualitative Inquiry.

