# Supplementary material for: Remote care in UK general practice: baseline data on 11 case studies
Source: NIHR Open Res. 2022 Nov 29;2:47. Originally published 2022 Aug 8. [Version 2] doi: 10.3310/nihropenres.13290.2 (PMC7614213; doi:10.3310/nihropenres.13290.2)
Supplement: Peer review history

# Reviewer report: Reviewer response for version 2

**DOI:** 10.3310/nihropenres.14479.r28880

**Published:** 2022-12-06

**Authors:** Anthony (Tony) Dowell

**Affiliations:** 1 Department of Primary Health Care and General Practice, University of Otago Wellington, Wellington, New Zealand

I thank the author for their thoughtful and considered responses to review. This is an important ongoing area of research for Primary Care.

Is the work clearly and accurately presented and does it cite the current literature?

Yes

If applicable, is the statistical analysis and its interpretation appropriate?

Yes

Are all the source data underlying the results available to ensure full reproducibility?

Yes

Is the study design appropriate and is the work technically sound?

Yes

Are the conclusions drawn adequately supported by the results?

Yes

Are sufficient details of methods and analysis provided to allow replication by others?

Yes

Reviewer Expertise:

Evaluation of COVID response. Complexity and Implementation science in primary care.

I confirm that I have read this submission and believe that I have an appropriate level of expertise to confirm that it is of an acceptable scientific standard.

# Reviewer report: Reviewer response for version 1

**DOI:** 10.3310/nihropenres.14414.r28789

**Published:** 2022-11-08

**Authors:** Jennifer Newbould

**Affiliations:** 1 Cambridge Centre for Health Services Research, RAND Europe, Cambridge, UK

This article reports on a mixed methods, primarily qualitative study, which seeks to study general practices as they balance remote and in-person care in the period following the COVID-19 pandemic. The plain English summary provides a concise and appropriately detailed account of the research with appropriate use of lay language and explanation of more technical terms. The introduction provides good background to the topic area and context within which the data were gathered. In the methods section the authors detail the conduct of the study, with appropriate reference to other documents should the reader require more detailed information. The approach it outlined in detail, including the composition of the team, a point of great importance with the researcher in resistance approach. The results section provides detailed information on the participating practices which provides important context to the findings presented. The clarification of practices by the digital maturity matrix is a helpful way of contextualising where on a spectrum practices are. There are good use of tables and figures throughout this paper which provide detailed information to the reader which enhances the main text. In the discussion section an appropriate summary is provided and themes to be explored further in the study outlined.

 In relation to points where further clarification from the authors might be sought:

- I was unclear if the practice names are actual names or pseudonyms? Clarification on this point might usefully be added to the text.
- The research is described as mixed methods, primarily qualitative yet this paper describes only qualitative approaches – where there also quantitative elements? Or are these at other time points in the programme of work?
- It would be useful to clarify for the reader some information on the depth of fieldwork undertaken to inform this paper, as well as the number of interviews conducted.
- In table 1 the authors identify formal and informal interviews, it would be good to explain within the text how these differ from each other.
- The results (under innovation and digital maturity) refer to a familiarisation process – it would be beneficial to provide detail to the reader as to what this includes.
- The sample of 11 practices display characteristics which make the ‘non typical’ of general practices across England - I feel this caveat to data should be made more explicitly in the discussion section.

Overall, I find this work to be of good quality and endorse the work is clearly presented, of sound study design and the conclusions adequately supported by the results. At a time of enormous change in general practice this feels like a timely and much needed research study, the design of which enables longitudinal and in-depth understanding of a range of practices as they balance remote and in-person care. I wish the authors all the best with the study.

Is the work clearly and accurately presented and does it cite the current literature?

Yes

If applicable, is the statistical analysis and its interpretation appropriate?

Not applicable

Are all the source data underlying the results available to ensure full reproducibility?

Yes

Is the study design appropriate and is the work technically sound?

Yes

Are the conclusions drawn adequately supported by the results?

Yes

Are sufficient details of methods and analysis provided to allow replication by others?

Yes

Reviewer Expertise:

Primary care research focused on non face-to-face approaches

I confirm that I have read this submission and believe that I have an appropriate level of expertise to confirm that it is of an acceptable scientific standard.

# Response

**Published:** 2022-11-21

**Authors:** Trisha Greenhalgh

**Affiliations:** University of Oxford, UK

Jennifer Newbould (R2)

 This article reports on a mixed methods, primarily qualitative study, which seeks to study general practices as they balance remote and in-person care in the period following the COVID-19 pandemic. The plain English summary provides a concise and appropriately detailed account of the research with appropriate use of lay language and explanation of more technical terms. The introduction provides good background to the topic area and context within which the data were gathered. In the methods section the authors detail the conduct of the study, with appropriate reference to other documents should the reader require more detailed information. The approach it outlined in detail, including the composition of the team, a point of great importance with the researcher in resistance approach. The results section provides detailed information on the participating practices which provides important context to the findings presented. The clarification of practices by the digital maturity matrix is a helpful way of contextualising where on a spectrum practices are. There are good use of tables and figures throughout this paper which provide detailed information to the reader which enhances the main text. In the discussion section an appropriate summary is provided and themes to be explored further in the study outlined.

 In relation to points where further clarification from the authors might be sought:

- I was unclear if the practice names are actual names or pseudonyms? Clarification on this point might usefully be added to the text.

**Pseudonyms! Added (page 10).**

- The research is described as mixed methods, primarily qualitative yet this paper describes only qualitative approaches – where there also quantitative elements? Or are these at other time points in the programme of work?

**Quant data are descriptive only (e.g. number of patients on list, waiting times). Explained, page 9.**

- It would be useful to clarify for the reader some information on the depth of fieldwork undertaken to inform this paper, as well as the number of interviews conducted.

**This was summarised in Table 1 but we’ve also added a sentence in the main text, page 9.**

- In table 1 the authors identify formal and informal interviews, it would be good to explain within the text how these differ from each other.

**This is explained in detail in the protocol paper but added briefly on page 9-10.**

- The results (under innovation and digital maturity) refer to a familiarisation process – it would be beneficial to provide detail to the reader as to what this includes.

**Added, page 9.**

- The sample of 11 practices display characteristics which make the ‘non typical’ of general practices across England - I feel this caveat to data should be made more explicitly in the discussion section.

**Added, page 30, but see above – I think we may have over-emphasised this. Actually I think the spread of practices is pretty typical with the exception that we failed to recruit practices who were actually already on their knees from the current NHS pressures. We got a few who weren’t far from that state though.**

 Overall, I find this work to be of good quality and endorse the work is clearly presented, of sound study design and the conclusions adequately supported by the results. At a time of enormous change in general practice this feels like a timely and much needed research study, the design of which enables longitudinal and in-depth understanding of a range of practices as they balance remote and in-person care. I wish the authors all the best with the study.

**Thanks! We build on your work of course.**

# Reviewer report: Reviewer response for version 1

**DOI:** 10.3310/nihropenres.14414.r28742

**Published:** 2022-08-17

**Authors:** Anthony (Tony) Dowell

**Affiliations:** 1 Department of Primary Health Care and General Practice, University of Otago Wellington, Wellington, New Zealand

As stated by the authors in the abstract, ‘virtual care’, became the prime form of providing primary care, during the early stages of the corona virus pandemic, providing opportunities for technology innovation and also challenges for both patients and health providers in maintaining effective clinical relationships and care. As restrictions on face to face health interactions have been relaxed, health systems are weighing up the optimal balance between virtual and ‘in person’ contact.

 Primary care and General Practice are notable for the variation in working style and practice across both individual practitioners and practices. This study with its depth longitudinal qualitative case study and co-design approach has the potential to provide valuable insights as to how this balance of different means of care will be negotiated.

**Abstract:** 

- The abstract provides a clear and concise review of the background and methodology chosen to answer the research questions.

**Introduction:** 

- The introduction provides a clear background to the UK general practice response to the need for a ‘digital response’ at the start of the COVID-19 pandemic, and provides appropriate descriptors of the different types of virtual care offered.
- It would be helpful (if available), particularly for international readers, to give some indication of the balance of digital portal and video / telephone consult use prior to the pandemic.
- The introduction appropriately highlights the importance of equity considerations in the provision of digital care, and questions to view that digital equates to efficient care delivery. The authors make a compelling case for both the need for their study and the chosen methodology by highlighting the absence of detailed in depth and highly granular case studies to explore how different practices will navigate the new ‘hybrid’ environment.

**Methods:** 

- The overall mainly qualitative methodology is appropriate to answer the research questions. Particular strengths of the methodology are the use of the ‘researcher in residence’ in stream one and the co-design elements of workstream 2.
- The paper states that most of the interaction with the practice team was via phone or video link, which seems to imply the researcher in residence was not able to observe many of the day to day workings of the practice. Was this the case?
- Table 1 is a useful graphical summary to the aims and methodology. 
- There is variation in the interview sampling frame in each practice. Was there contact with a representative with all those who would be undertaking virtual health consultations in each practice. E.g., at least one nursing interview?
- The use of additional information sources and document analysis adds to the rigour of the methods.
- It is clear that the research team has appropriate training in the chosen research methods. To what extent were there specific group training sessions for the whole team, such that there were consistent practice overviews?
- The use of the two senior researchers providing a synthesised overarching narrative and feedback on the familiarisation documents is a useful addition to the methods.

**Results: **

- The results and major findings are clearly defined and displayed.
- The Table 2 summary is very helpful.
- While the protocol paper contains details of the digital maturity scale, it may be helpful to give a brief idea, particularly for international readers, what the different levels mean. It would be helpful to indicate at the start that Table 3 gives and indication of these levels.
- The use of widespread geographical variation is a positive feature of the study. Given the multiple commentaries about the 'North / South' divide, was there any reason why there were no North of England practices in the study?
- The practice selection descriptions do seem to suggest  ‘all are more outward-looking than average’. Was there any attempt made, either by incentivisation or other means, to try and recruit more ‘average’ practices to the study?
- The initial finding of practice commitment to the local population is an important finding from an appreciative inquiry perspective. This could perhaps be highlighted more prominently?
- I am not entirely clear from the results how quickly pre-pandemic preparedness was translated into a digital COVID response or whether the levels of digital maturity in Table 3 represents largely their digital status prior to COVID? Interview data was collected between October 2021 and June 2022; were interviews and data intended to assess digital opportunities and challenges during that time frame?
- The results section flows well in a logical sequence from the description of the different capacity and capability to the cross cutting themes.
- The results describe primary care services under pressure (flooding metaphors) and the pressure of having to respond digitally. It would be helpful to make clear whether there was any reduction in overall workload from patients staying away because of COVID concerns or other reasons, hence freeing up clinician time to undertake digital consultations? This has been noted in other settings, with one concern being potential harm because patients delayed seeking usual care.
- The cross cutting themes are well described with appropriate focus on access, quality and access.
- There is a wealth of detail in the cross cutting themes. It might be helpful to have a box / table with a list of the overview themes and brief descriptor at the outset to guide the reader through this section.

**Discussion: **

- The discussion provides a concise summary of findings and then places further themes for exploration in context.
- The discussion themes are well written. There is no external referencing in the discussion either with other local UK or international commentaries. Is there a reason for this, given the comparative literature on such themes as digital response to COVID, co-design and equity in these areas?

Is the work clearly and accurately presented and does it cite the current literature?

Yes

If applicable, is the statistical analysis and its interpretation appropriate?

Yes

Are all the source data underlying the results available to ensure full reproducibility?

Yes

Is the study design appropriate and is the work technically sound?

Yes

Are the conclusions drawn adequately supported by the results?

Yes

Are sufficient details of methods and analysis provided to allow replication by others?

Yes

Reviewer Expertise:

Evaluation of COVID response. Complexity and Implementation science in primary care.

I confirm that I have read this submission and believe that I have an appropriate level of expertise to confirm that it is of an acceptable scientific standard, however I have significant reservations, as outlined above.

# Response

**Published:** 2022-11-21

**Authors:** Trisha Greenhalgh

**Affiliations:** University of Oxford, UK

Tony Dowell (R1)

 As stated by the authors in the abstract, ‘virtual care’, became the prime form of providing primary care, during the early stages of the corona virus pandemic, providing opportunities for technology innovation and also challenges for both patients and health providers in maintaining effective clinical relationships and care. As restrictions on face to face health interactions have been relaxed, health systems are weighing up the optimal balance between virtual and ‘in person’ contact.

 Primary care and General Practice are notable for the variation in working style and practice across both individual practitioners and practices. This study with its depth longitudinal qualitative case study and co-design approach has the potential to provide valuable insights as to how this balance of different means of care will be negotiated.

**Abstract:** 

- The abstract provides a clear and concise review of the background and methodology chosen to answer the research questions.

**Introduction:** 

- The introduction provides a clear background to the UK general practice response to the need for a ‘digital response’ at the start of the COVID-19 pandemic, and provides appropriate descriptors of the different types of virtual care offered.
- It would be helpful (if available), particularly for international readers, to give some indication of the balance of digital portal and video / telephone consult use prior to the pandemic.

**This has been done (page 8). Answer: In January 2020 14.7% of primary care consultations were by phone; in April 2020 this had risen to 50.6% and by October 2020 had fallen again to 37%. The absolute number of consultations fell dramatically in the early weeks of the pandemic: from 22 million per week in Jan 2020 to 15 million in April – but rose to almost 27 million in October. Video and e-consultations accounted for a tiny percentage pre-pandemic and didn’t increase much percentage-wise during the pandemic.**

**In England, for example, video and e-consultations combined accounted for fewer than 0.5% of general practice consultations in December 2021**

- The introduction appropriately highlights the importance of equity considerations in the provision of digital care, and questions to view that digital equates to efficient care delivery. The authors make a compelling case for both the need for their study and the chosen methodology by highlighting the absence of detailed in depth and highly granular case studies to explore how different practices will navigate the new ‘hybrid’ environment.

**Methods:** 

- The overall mainly qualitative methodology is appropriate to answer the research questions. Particular strengths of the methodology are the use of the ‘researcher in residence’ in stream one and the co-design elements of workstream 2.
- The paper states that most of the interaction with the practice team was via phone or video link, which seems to imply the researcher in residence was not able to observe many of the day to day workings of the practice. Was this the case?

**It was for the first few weeks when COVID-19 restrictions were in place, which is where we were when the baseline findings paper was submitted. More recent data on all practices includes direct ethnography. We’ve clarified on page 9 and 32.**

- Table 1 is a useful graphical summary to the aims and methodology. 
- There is variation in the interview sampling frame in each practice. Was there contact with a representative with all those who would be undertaking virtual health consultations in each practice. E.g., at least one nursing interview?

**No. See Table 1. In some practices (e.g. Queens Road, which came on board a bit later than all the others), we’d only done ‘informal’ interviews at this stage. We got who we could, as practices were so busy. However, many of these interviews were rich and detailed, and we got a good picture of what the issues were. We wanted to publish this baseline data even though incomplete, precisely because it forms the baseline against which we have followed their progress.**

- The use of additional information sources and document analysis adds to the rigour of the methods.
- It is clear that the research team has appropriate training in the chosen research methods. To what extent were there specific group training sessions for the whole team, such that there were consistent practice overviews?

**Not so much group training sessions but weekly or fortnightly ‘huddles’ – online themed discussion sessions where we learn what’s going on in the other practices and what approaches are being used. Added, page 11.**

- The use of the two senior researchers providing a synthesised overarching narrative and feedback on the familiarisation documents is a useful addition to the methods.

**Results: **

- The results and major findings are clearly defined and displayed.
- The Table 2 summary is very helpful.
- While the protocol paper contains details of the digital maturity scale, it may be helpful to give a brief idea, particularly for international readers, what the different levels mean. It would be helpful to indicate at the start that Table 3 gives and indication of these levels.

**The problem here is we’re going to be repeating the protocol paper if we add detailed explanations about the digital maturity scale. We already have this brief explanation: “… digital maturity using the following five-point scale ^2^ : • (traditional – few or no digital innovations or strategy), •• (traditional with lone innovator – one person keen and attempting to introduce digital innovations and services), ••• (digitally curious – experimenting with digital innovations but not planning or implementing these strategically), •••• (digitally strategic – investing in digital innovations and services, and in some cases strategically *disinvesting* in them) and ••••• (system-oriented – confidently providing a range of digital services and seeking to support others to do the same).”**

**Note to reviewer: the protocol paper and the baseline findings paper are intended to be read as a pair. They are both published on NIHR Open site and will be linked to each other. We wanted to name the same reviewers for both papers but NIHR Open didn’t allow this because of a blanket rule that the reviewers should be “independent”. This makes no sense of course in our case but we don’t make the rules!**

- The use of widespread geographical variation is a positive feature of the study. Given the multiple commentaries about the 'North / South' divide, was there any reason why there were no North of England practices in the study?

**Just coincidence really. We’re just applying for another grant to extend RBD2 and we’ve added a site in Birmingham. I guess the point is, Glasgow and South Wales are also pretty deprived and remote Wales also has other features we wanted to sample (e.g. remoteness! And the Welsh language).**

- The practice selection descriptions do seem to suggest  ‘all are more outward-looking than average’. Was there any attempt made, either by incentivisation or other means, to try and recruit more ‘average’ practices to the study?

Point taken, but I wonder if we over-egged this point in the initial draft. All we’re saying is that in the current climate (NHS general practice is *on its knees*), ANY practice that agrees to participate in this kind of research is probably atypical. It would of course be impossible to recruit a practice that is too busy to participate (and that’s probably most of them). So the only way round this is to interpret the findings in the light of the likely skew. There is however huge diversity in practice characteristics – small/large, affluent/deprived, digitally mature/immature, urban/rural etc etc. I don’t think we should over-interpret this sentence “practices were more outward-looking than average”.  Ethics rules would not allow us to “incentivise” (e.g. pay more than the going rate) to induce participation. 

- The initial finding of practice commitment to the local population is an important finding from an appreciative inquiry perspective. This could perhaps be highlighted more prominently?

**Possibly, but I also wonder if it’s just something that all GP practices say? We’ll bear it in mind as we follow them further!**

- I am not entirely clear from the results how quickly pre-pandemic preparedness was translated into a digital COVID response or whether the levels of digital maturity in Table 3 represents largely their digital status prior to COVID? Interview data was collected between October 2021 and June 2022; were interviews and data intended to assess digital opportunities and challenges during that time frame?

Digital COVID response from UK general practice was staggeringly fast—happened over 3 weeks in March-April 2020. The shift was threefold: a) ‘total triage’ (patients had to book online or wait their turn to have a receptionist help them by phone); b) vast majority of consultations by telephone; c) video consultations introduced using novel (fast-developed) technologies. But the first two of these didn’t require much in the way of new tech. What was more important in digital maturity terms was the practice’s *strategic* approach to technologies in general. Was there a budget line for technologies in the annual business plan? Were technologies purchased and evaluated *strategically* to pursue particular goals (or were they the pet gadgets of the techy partner)? Was there a training budget for staff to be taught to use tech? Was the practice’s IT infrastructure adequate (e.g. server power)? Was there a member of staff around who knew how to install stuff and could parley with helpdesk people? So it’s not just ‘did they do video consultations?’ but ‘were they innovative technologically [including acknowledging that tech needs training and support and routinization]?’.  To answer the question, digital maturity mostly reflects the status prior to covid (we’ve clarified this on page 12). None of the practices experienced fundamental transformation during covid, they just switched to online booking and phone call-backs!

- The results section flows well in a logical sequence from the description of the different capacity and capability to the cross cutting themes.
- The results describe primary care services under pressure (flooding metaphors) and the pressure of having to respond digitally. It would be helpful to make clear whether there was any reduction in overall workload from patients staying away because of COVID concerns or other reasons, hence freeing up clinician time to undertake digital consultations? This has been noted in other settings, with one concern being potential harm because patients delayed seeking usual care.

**Yes massively, but only for a few months in 2020. Added (page 8). (plus please see response to R2)**

- The cross cutting themes are well described with appropriate focus on access, quality and access.
- There is a wealth of detail in the cross cutting themes. It might be helpful to have a box / table with a list of the overview themes and brief descriptor at the outset to guide the reader through this section.

**Great suggestion. Added, page 21-22.**

**Discussion: **

- The discussion provides a concise summary of findings and then places further themes for exploration in context.
- The discussion themes are well written. There is no external referencing in the discussion either with other local UK or international commentaries. Is there a reason for this, given the comparative literature on such themes as digital response to COVID, co-design and equity in these areas?

**There was no reason! Some external references now added (thanks for the prompt!).**
